# New population-based exome data question the pathogenicity of some genetic variants previously associated with Marfan syndrome

**DOI:** 10.1186/1471-2156-15-74

**Published:** 2014-06-18

**Authors:** Ren-Qiang Yang, Javad Jabbari, Xiao-Shu Cheng, Reza Jabbari, Jonas B Nielsen, Bjarke Risgaard, Xu Chen, Ahmad Sajadieh, Stig Haunsø, Jesper Hastrup Svendsen, Morten S Olesen, Jacob Tfelt-Hansen

**Affiliations:** 1 Laboratory of Molecular Cardiology, Department of Cardiology, the Heart Centre, Copenhagen University Hospital, Rigshospitalet, Copenhagen, Denmark; 2 The Danish National Research Foundation Centre for Cardiac Arrhythmia, Copenhagen, Denmark; 3 Department of Cardiology, Institute of Cardiovascular Disease, the Heart Centre, the Second Affiliated Hospital, Nanchang University, Nanchang, China; 4 Department of Cardiology, Copenhagen University Hospital of Bispebjerg, Bispebjerg, Denmark; 5 Department of Medicine and Surgery, Faculty of Health and Medical Science, University of Copenhagen, Copenhagen, Denmark

**Keywords:** Marfan syndrome, Genetic testing, HGMD, The NHLBI GO exome sequencing project, Variant

## Abstract

**Background:**

Marfan syndrome (MFS) is a rare autosomal dominantly inherited connective tissue disorder with an estimated prevalence of 1:5,000. More than 1000 variants have been previously reported to be associated with MFS. However, the disease-causing effect of these variants may be questionable as many of the original studies used low number of controls. To study whether there are possible false-positive variants associated with MFS, four *in silico* prediction tools (*SIFT, Polyphen-2*, *Grantham score*, and *conservation across species*) were used to predict the pathogenicity of these variant.

**Results:**

Twenty-three out of 891 previously MFS-associated variants were identified in the ESP. These variants were distributed on 100 heterozygote carriers in 6494 screened individuals. This corresponds to a genotype prevalence of 1:65 for MFS. Using a more conservative approach (cutoff value of >2 carriers in the EPS), 10 variants affected a total of 82 individuals. This gives a genotype prevalence of 1:79 (82:6494) in the ESP. A significantly higher frequency of MFS-associated variants not present in the ESP were predicted to be pathogenic with the agreement of ≥3 prediction tools, compared to the variants present in the ESP (p = 3.5 × 10^−15^).

**Conclusions:**

This study showed a higher genotype prevalence of MFS than expected from the phenotype prevalence in the general population. The high genotype prevalence suggests that these variants are not the monogenic cause of MFS. Therefore, caution should be taken with regard to disease stratification based on these previously reported MFS-associated variants.

## Background

Marfan syndrome (MFS; OMIM 154700), first described by Antoine Marfan in 1898, is an autosomal dominantly inherited connective tissue disorder with a phenotype that involves mainly the cardiovascular, ocular, and skeletal systems. The prevalence of MFS in the general US and European population has been estimated to be 1:5,000 [1,2]. MFS has a high penetrance, but variable expression [3]. Cardiovascular complications are the main cause of premature death among MFS patients [4,5]. Besides aortic aneurysm and/or dissection, MFS can lead to valvular heart disease [6], enlargement of the proximal pulmonary artery [7], congestive heart failure [8], and arrhythmias [9].

According to the current “revised Ghent nosology”, the diagnosis of MFS should be based on clinical manifestation, family history, and molecular genetic testing of the fibrillin 1 gene (*FBN1* gene) and the clinical criteria employs a set of manifestations in many tissues [10]. Approximately 25% of all MFS patients do not have a family history and hence, represents new cases due to de novo mutations [3]. To date, more than 1000 variants have been reported to be associated with MFS [11]. Recently, transforming growth factor beta (TGFB) has been found to play a pivotal role in the progression of MFS [12]. A disease that has many similarities with Marfan syndrome, termed Loeys-Dietz syndrome, was identified to be caused by variants in the transforming growth factor-beta type II receptor (*TGFBR2*) [13]. An atypical or incomplete MFS has also been described in some patients caused by genetic variants in transforming growth factor-beta type I receptor (*TGFBR1*) and *TGFBR2*[14]. However, to our knowledge, there are no systematic studies focusing on separating genetic noise from disease-causing variations by identifying variants previously associated with MFS in large-scale populations.

Until recently, there has only been little knowledge regarding the distribution of genetic variations in general population, especially with regard to low-frequency variants of MFS. This is potentially a problem, when rare variants are associated with MFS because of the risk of false-positive findings. The disease-causing role of some of these variants is questionable as many of these studies have used low number of controls. This problem has now partly been solved with the release of exome data from the NHLBI GO Exome Sequencing Project (ESP). Large-scale surveys of human genetic variations provide an important chance to identify causative variants, notable for an excess of rare genetic variants [15].

The aim of this study was to identify false positive variants previously associated with Marfan syndrome. This is important since genetic testing today is used in order to confirm MFS diagnose according to the revised Ghent Nosology. In the absence of family history and aortic root dilatation/dissection, but presence of ectopialentis, the identification of an FBN1 variant previously associated with aortic disease is required in making the diagnosis. Also in the absence of family history and ectopialentis, but presence of aortic root dilatation/dissection a genetic test for identification of mutation in FBN1 is sufficient to establish a MFS diagnosis. Due to the importance of a positive genetic finding in patients suspected of MFS, identification of false positive variants has major clinical implications. Furthermore, we aimed to provide comprehensive *in silico* prediction analysis to all MFS-associated variants, in order to better classify the impact of the variations on the encoded proteins.

## Methods

In the ESP, next-generation sequencing was carried out for all protein-coding regions in 6,503 individuals from different population studies [16]. It currently contains 2,203 unrelated African-Americans (AA) and 4,300 unrelated European-Americans (EA) (13,006 alleles in total). In the ESP database, samples were selected to contain healthy controls, the extremes of specific traits (LDL and blood pressure), and specific diseases (early onset myocardial infarction and early onset stroke), and lung diseases. To our knowledge, patients with MFS have not been included intentional in ESP. Clinical data were not available, nor on request.

To find all genes and variants associated with MFS, a search for missense and nonsense variants was performed in the Human Gene Mutation Database (HGMD Professional 2013.2) [17]. Additionally, the following literature search query was used in the PubMed database ((Marfan) OR (Marfan syndrome) or (“Marfan syndrome” [Mesh])) AND ((Genetic) OR (“Genetics” [Mesh])) AND ((mutation) OR (variant)). In this way, we included 3 variants recently associated with MFS [18-20]. Finally, we searched the ESP for all these variants (Version: v.0.0.20. (June 7, 2013)). We used a terminology so that MFS-associated variants identified in the ESP database are termed ESP-Positive variants and variants not identified in ESP as ESP-Negative variants. Because of lack of data regarding introns and untranslated regions in the ESP, variants in introns and untranslated regions could not be included. Furthermore, we did not include variants in the genes *COL1A2* and *LTBP2*, since variants in these genes have not been convincingly associated with MFS. Since the aim of this study was to include all possible variants previously associated with MFS, we also included variants which were associated with MFS using the old Ghent criteria.

Based on the phenotype prevalence of MFS (1/5000 = 0.02%), the expected prevalence of MFS in the ESP population is 0.02% (95% CI 0.0%-0.05%) for 1.3 subjects out of 6503.

Therefore, estimated number of individuals affected by MFS in the ESP can be expected to be no more than 2. That is, a given variant with complete penetrance should theoretically not affect more than 2 ESP alleles in order to be the cause of a monogenic form of MFS. For a conservative approach, we therefore used a cutoff value of >2 affected the ESP alleles, to estimate the genotype prevalence of MFS.

The literature was searched for functional data and familial co-segregation of all the MFS-associated ESP-Positive variants. Familial co-segregation was defined as at least two family members having the same genotype as well as the same MFS phenotype.

Four traditional prediction tools (*SIFT,Polyphen-2*, *Grantham score*, and *conservation across species*) were applied in order to predict the pathogenicity of all MFS-associated missense variants. Nonsense variants that were assumed “probably damaging” were not included in the prediction analyses. Missense variants were classified as damaging if they were predicted to be damaging by ≥3 of the four applied prediction tools [21]. Conservation across species of sequence indicates that a particular genotype have been preserved during the evolution. For more detail, please see online Additional file 1. The list of ESP-Negative variants is shown in the Additional file 2: Table S1. Any difference in the proportion of variants predicted to be damaging for ESP-Positive variants compared with ESP-Negative variants was assessed with the Fisher’s exact. A two-tailed P-value <0.05 was considered statistical significant.

## Results

### Variants associated with MFS

Three genes have been previously reported to be involved in MFS: *FBN1, TGFBR1*, and *TGFBR2*. In *FBN1, TGFBR1*, and *TGFBR2*, a total of 891 missense/nonsense variants have been reported to be associated with MFS. There are737missense and 154 nonsense variants associated with MFS. Overall, 97% (861/891) of all variants were found in *FBN1*, less than 3% (26/891) and less than 1% (4/891) was found in *TGFBR2*and *TGFBR1,* respectively.

### The ESP-positive variants

Twenty-three out of the 891 variants previously associated with MFS were identified in the ESP population. All of the 23ESP-positive variants were missense variants found in the *FBN1* gene, and 87% (20/23) of these variants have been reported as novel mutations in the published original papers. In total, 100 heterozygote carriers of these 23variants were present in the ESP. The *FBN1* gene was screened in 6,494 individuals on average in the ESP. This corresponds to a genotype prevalence of 1:65 (100:6494) in the ESP (Table 1). This is a very large overrepresentation of MFS associated variants; hence, it is likely that many of these variants are not the major/monogenic cause of MFS.

**Table 1:** Variants previously associated with Marfan syndrome difference distribution of one allele in the ESP population

**Gene**	**Variant**	**Amino acid**	**European Americans genotype**			**African Americans genotype**			**All genotype**			**Ref**
			**Minor/Minor**	**Minor/Major**	**Major/Major**	**Minor/Minor**	**Minor/Major**	**Major/Major**	**Minor/Minor**	**Minor/Major**	**Major/Major**	
	c.59A > G^#^	p.Y20C	0	3	4293	0	0	2197	0	3	6490	[1]
	c.1027G > A^#^	p.G343R	0	2	4294	0	0	2197	0	2	6491	[22]
	c.1345G > A^#^	p.V449I	0	2	4294	0	0	2197	0	2	6491	[23]
	c.2056G > A^#^	p.A686T	0	0	4296	0	1	2196	0	1	6492	[24]
	c.2927G > A^#^	p.R976H	0	2	4294	0	0	2198	0	2	6492	[25]
	c.3058A > G^#^	p.T1020A	0	3	4293	0	0	2198	0	3	6491	[26]
	c.3422C > T	p.P1141L	0	13	4283	0	1	2197	0	14	6480	[27]
	c.3509G > A^#^	p.R1170H	0	23	4273	0	2	2196	0	25	6469	[28]
	c.3797A > T^#^	p.Y1266F	0	4	4292	0	0	2198	0	4	6490	[29]
	c.3845A > G^#^	p.N1282S	0	2	4294	0	1	2197	0	3	6491	[30]
	c.4270C > G	p.P1424A	0	4	4292	0	0	2198	0	4	6490	[31]
***FBN1***	c.6055G > A	p.E2019K	0	1	4295	0	0	2198	0	1	6493	[32]
	c.6700G > A^#^	p.V2234M	0	8	4288	0	0	2198	0	8	6486	[22]
	c.7241G > A^#^	p.R2414Q	0	1	4295	0	0	2198	0	1	6493	[33]
	c.7379A > G^#^	p.K2460R	0	2	4294	0	0	2198	0	2	6492	[34]
	c.7660C > T^#^	p.R2554W	0	1	4295	0	0	2198	0	1	6493	[25]
	c.7661G > A^#^	p.R2554Q	0	1	4295	0	0	2198	0	1	6493	[32]
	c.7702G > A^#^	p.V2568M	0	0	4296	0	1	2197	0	1	6493	[34]
	c.7846A > G^#^	p.I2616V	0	3	4293	0	1	2197	0	4	6490	[34]
	c.7852G > A^#^	p.G2618R	0	1	4295	0	1	2197	0	2	6492	[29]
	c.8081G > A^#^	p.R2694Q	0	1	4295	0	0	2198	0	1	6493	[24]
	c.8176C > T^#^	p.R2726W	0	9	4287	0	5	2193	0	14	6480	[35]
	c.8494A > G^#^	p.S2832G	0	1	4295	0	0	2198	0	1	6493	[24]

ESP: Exome Sequencing Project. ^#^The variant was reported as a novel variant in the paper.

Using a more conservative approach (cutoff value of >2 present in the EPS), 10 out of the 23 variants were present in three or more individuals in the ESP. These variants affected a total of 82 individuals giving a genotype prevalence of 1:79 (82:6494). The remaining 13 variants are rare non-synonymous variants (<=2), affecting 18 individuals in the ESP, giving a genotype prevalence of 1:361 (18:6494) (Table 1).

Among all *FBN1* MFS-associated variants, only 3% (23/861) were present in the ESP. Family co-segregation and functional characterization data of all the 23 variants are present in Table 2. Most of MFS-associated variants were ESP-Negative variants. None of MFS- associated variants in *TGFBR1* and *TGFBR2* was identified in the ESP population. Furthermore, clinical data for these 23 patients is shown in Additional file 3: Table S2.

**Table 2:** Functional data and family co-segregation for genes and variants in the ESP population

**Gene**	**Variant**	**dbSNP**	**All genotypes (EA + AA)**	**SIFT**	**PolyPhen-2**	**Grantham score**	**Conservation**	**Agreement**	**HGMD**	**Family**	**Functional data**
							**YES/NO**	≥**3**	**Variant class**	**Co-segregation**	
	c.59A > G	rs201309310	CC = 0/CT = 3/TT = 6490	Tolerant	Benign	D (194)	YES	B	DM	YES	NO
	c.1027G > A	rs146726731	TT = 0/TC = 2/CC = 6491	Tolerant	Possibly damaging	D (125)	YES	D	DM	NO	NO
	c.1345G > A	rs139058991	TT = 0/TC = 2/CC = 6491	Tolerant	Benign	B (29)	YES	B	DM	NO	NO
	c.2056G > A	rs377621293	TT = 0/TC = 1/CC = 6492	Tolerant	Benign	B (58)	YES	B	DM	NO	NO
	c.2927G > A	rs140954477	TT = 0/TC = 2/CC = 6492	Tolerant	Probably damaging	B (29)	YES	B	DM	NO	NO
	c.3058A > G	rs111801777	CC = 0/CT = 3/TT = 6491	Tolerant	Benign	B (58)	NO	B	DM	NO	NO
	c.3422C > T	rs2228241	AA = 0/AG = 14/GG = 6480	Damaging	Probably damaging	B (98)	NO	B	DM?	NO	NO
	c.3509G > A	rs137854475	TT = 0/TC = 25/CC = 6469	Tolerant	Benign	B (29)	YES	B	DM	YES	NO
	c.3797A > T	rs200283837	AA = 0/AT = 4/TT = 6490	Tolerant	Benign	B (22)	NO	B	DM	NO	NO
	c.3845A > G	rs140647	CC = 0/CT = 3/TT = 6491	Damaging	Benign	B (46)	YES	B	DM?	NO	NO
	c.4270C > G	rs201273753	CC = 0/CG = 4/GG = 6490	Damaging	Probably damaging	B (27)	NO	B	DM	NO	NO
***FBN1***	c.6055G > A	rs377149130	TT = 0/TC = 1/CC = 6493	Tolerant	Possibly damaging	B (56)	NO	B	DM?	YES	NO
	c.6700G > A	rs112084407	TT = 0/TC = 8/CC = 6486	Tolerant	Benign	B (21)	NO	B	DM	NO	NO
	c.7241G > A	rs143863014	TT = 0/TC = 1/CC = 6493	Tolerant	Benign	B (43)	NO	B	DM	YES	NO
	c.7379A > G	rs144189837	CC = 0/CT = 2/TT = 6492	Tolerant	Possibly damaging	B (26)	YES	B	DM	NO	NO
	c.7660C > T	rs369294972	AA = 0/AG = 1/GG = 6493	Damaging	Probably damaging	D (101)	YES	D	DM	YES	NO
	c.7661G > A	rs199522781	TT = 0/TC = 1/CC = 6493	Tolerant	Probably damaging	B (43)	YES	B	DM?	NO	NO
	c.7702G > A	rs138558987	TT = 0/TC = 1/CC = 6493	Damaging	Benign	B (21)	YES	B	DM	NO	NO
	c.7846A > G	rs143677764	CC = 0/CT = 4/TT = 6490	Tolerant	Benign	B (29)	NO	B	DM	YES	NO
	c.7852G > A	rs141133182	TT = 0/TC = 2/CC = 6492	Tolerant	Probably damaging	D (125)	YES	D	DM	NO	NO
	c.8081G > A	rs371375126	TT = 0/TC = 1/CC = 6493	Tolerant	Benign	B (43)	YES	B	DM	NO	NO
	c.8176C > T	rs61746008	AA = 0/AG = 14/GG = 6480	Damaging	Benign	D (101)	NO	B	DM	YES	NO
	c.8494A > G	rs376933421	CC = 0/CT = 1/TT = 6493	Tolerant	Benign	B (56)	YES	B	DM	NO	NO

D: Damaging; B: Benign; DM: Disease causing mutation; ESP: Exome Sequencing Project; HGMD: Human Gene Mutation Database. EA: European Americans Genotype. AA: African Americans Genotype.

In order to test if the ESP data harbored an over representation, one variant *TGFBR2* V387M (rs35766612), previously reported to be involved in the patient with Marfanoid features [36], was genotyped in our own healthy Northern European control population (n = 704). There were 30 carriers of this variant in 6504 individuals on average in the ESP population. We found 4 carriers in our healthy population. The genotype prevalence of the variant in our healthy control population was comparable with that in the ESP (4:750 *vs*.30:6503, p = 0.578).

### Prediction analyses of the ESP-positive variants vs. the ESP-negative variants

**Figure 1:**
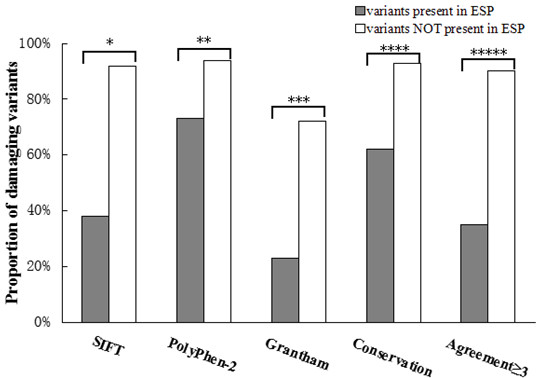
**Percentage of variants predicted to be pathogenic with four In silico tools prediction on variants present and not present in ESP database.** Differences in proportions of variants predicted to be damaging for those variants present in ESP versus variants not present in ESP were assessed using Fisher’s exact test. *p = 4.1 × 10^−10^, **p = 2.8 × 10^−11^, ***p = 1.1 × 10^−6^, ****p = 4.5 × 10^−5^, *****p = 3.5 × 10^−15^.

Using *SIFT* prediction, 26% (6/23) of the ESP-Positive variants were predicted to be damaging, compared with86% (613/713) of the ESP-Negative variants (p = 4.1 × 10^−10^). Using *Polyphen-2* prediction tool, the variants present in the ESP were predicted pathogenic in 39% (9/23) of the cases compared with 94% (670/713) of the ESP-Negative variants (p = 2.8 × 10^−11^). Using the *Grantham scores*, 22% (5/23) of the ESP-Positive variants were predicted to be damaging compared with 72% (516/713) of the ESP-Negative variants (p = 1.1 × 10^−6^). Finally, we used *Conservation across species* and found that 61% (14/23) of the ESP-Positive variants were identified in a conserved region, predicted as damaging, compared with 92% (659/713) of the ESP-Negative variants (p = 4.5 × 10^−5^). The calculation of the agreement of ≥3 pathogenicity predictions showed that 13% (3/23) of variants present in the ESP and 88% (627/713) of the ESP-Negative variants were predicted to be damaging (p = 3.5 × 10^−15^) (Figure 1). For more details, please see the online Additional file 2. Same trend was found when the ESP positive was divided into whether they occurred more or less than two times in the ESP (online Additional file 4: Figure S1).

## Discussion

The present study is the first to report and critically evaluate the genetic background noise in Marfan syndrome based on the prevalence of previously reported MFS-associated genetic variants in the ESP database. We found a much higher prevalence (1:65) of MFS-associated genetic variants in the ESP than expected according to the phenotype prevalence of 1:5,000 in the general US and European population. The ESP data is thought to be representative for this population.

In order to test if the ESP data harbored an over representation, one variant*TGFBR2* V387M (rs35766612), previously reported to be involved in the patient with Marfanoid features [36], was genotyped in our own healthy Northern European control population (n = 704). There were 30 carriers of this variant in 6,504 individuals on average in the ESP population. We found 4 carriers in our healthy population. The genotype prevalence of the variant in our healthy control population was comparable with that in the ESP (4:750 *vs*.30:6503, p = 0.578). For more details, please see the online Additional file 1. Recent papers have also established the prevalence of other rare variants genotyped in our Northern European control population and found that they were comparable to those of the ESP [37-39]. This is suggesting that it is unlikely that the ESP population harbor a major overrepresentation of some of the MFS-associated variants.

A prevalence like this is however unlikely to be caused by reduced penetrance or age-related delayed presentation of the disease, as MFS has a high penetrance with onset of symptoms early in life [3].

It is likely that some of these variants are either not a monogenic cause of MFS, or they have incomplete penetrance. Lucarini et al. [40] screened the *TGFBR1* gene in patients with MFS who were excluded as carriers of the variants in *FBN1* and *TGFBR2*. Their findings suggested that some variants are overrepresented in MSF patients compared to control suggesting that TGFBR1 may be the underlying genetic cause of MSF, but with low penetrance alleles in MFS.

Four common prediction tools were applied to evaluate the phylogenetic and physicochemical effects on the MFS-associated variants. Recently published data support the potential clinical utility of these tools [21]. In our study, we found that a much lower proportion of the ESP-Positive variants were predicted to be damaging compared with those ESP-Negative variants (13% *vs. *88%, p =3.5 × 10^−15^, Figure 1). This result further questions the pathogenic role of at least some of the variants present in the ESP. But it does not definitively exclude the possibility of pathogenicity. Some ESP-Positive variants in low frequency could potentially be disease causing. Accordingly, the presence of a variant in the ESP population does not exclude that the variant might be disease-causing, but is indeed questioning the variant disease causing potential, particularly when the variant presents in the ESP in high frequency. The same approach and concerns were recently also suggested in another disease; catecholaminergic polymorphic ventricular tachycardia [41].

We defined a cutoff value (>2) in the ESP (n = 6503) based on the expected prevalence of MFS (1:5,000). This cutoff is of course somewhat arbitrary because we do not know the exact prevalence of MFS in the ESP population. So assuming that MFS is a monogenic disorder, the findings of some variants with prevalence above the cutoff value of more than 2 in the ESP suggest that some of the variants at least may be false-positive, or incomplete penetrance. Reduced or incomplete penetrance is not uncommon in genetic diseases. It can be resulted from differential allelic expression or copy number variation [42]. The interpretation of variants with a prevalence below a certain cutoff value in the ESP may be considered monogenic disease-causing, disease-modifier or benign.

Genetic screening is gaining ground in the diagnostic workup of families and identification of populations at a high risk of suffering an inherited disease [43]. According to “the revised Ghent nosology”, genetic screening for variants may in some cases be the game changer in making the diagnose MFS [10].

Genetic screening for MFS in family members has become an important tool in family cascade screening. In particular, targeted testing of *FBN1* is recommended in cases where MFS is suspected following a cardiac examination. It is noteworthy, that only 23 out of 861 (3%) variants in *FBN1* were identified in the ESP. That is, 97% of the variants in *FBN1*were not among nearly 6,500 subjects in the ESP. This confirms the pivotal role and usefulness of the *FBN1* gene in genetic screening. Awareness of an *FBN1* variant should imply for increased vigilance for MFS. Treatment with an angiotensin receptor blocker has been proven to be effective in reducing rates of aortic root dilatation in MFS patients. So knowledge of an *FBN-1* variant may allow actionable interventions earlier in the natural history of the condition [5].

Lack of properly scaled control populations has always been a problem when dealing with low frequency genetic variations of rare monogenetic diseases. Without a reasonable control population we might misdiagnose family members undergoing genetic testing and follow-up. Based on our study, we strongly suggest that exome data, like the ESP, should be used as empirical data in research and clinical practice, alongside with known prediction tools to get a more exact understanding of the pathogenicity of the variants associated with MFS or other rare inherited disorders. It is important to keep in mind that the absence of variants in the ESP in itself, is not to be interpreted as the variant is disease causing, but certainly strengthen the possibility. Furthermore the Marfan-related mutations analyses in this study do not exclude that further potentially false-positive variants could be found in healthy persons in other populations such as Asians. Comparing findings in this study with other populations may reduce the rate of false positive variants.

## Conclusion

In this study we have identified 23 previously reported MFS-associated variants in the ESP database. The genotype prevalence of these variants corresponded to MFS prevalence of 1:65. The high genotype prevalence questions the causality of some of these variants, suggesting that these variants may not be the monogenic cause of MFS. Therefore, caution should be taken with regard to disease stratification based on variants previously associated with MFS.

## Competing interests

The authors declare that they have no competing interest.

## Authors’ contributions

RQY, JJ, XSC, RJ, JBN, BR, XC, AS, JHS, SH, MSO, and JT were involved in the data collection and analysis, and design of the study. RQY, JJ, MSO, and JT carried out the data analysis. JJ carried out statistical calculation. RQY, JJ, JBN, BR, MSO, and JT drafted the manuscript. All authors reviewed the manuscript and approved the final version.

## Supplementary Material

Additional file 1 The methods of prediction and genotyping a variant in Northern European control population. Click here for file

Additional file 2: Table S1 Variants associated with Marfan syndrome not present in ESP. Click here for file

Additional file 3: Table S2 Clinical data of FBN-1 ESP-positive variants. Click here for file

Additional file 4: Figure S1 Percentage of variants predicted to be pathogenic with four In silico tools prediction on variants present and not present in ESP database. Differences in proportions of variants predicted to be damaging for those variants present in ESP versus variants not present in ESP. Furthermore, variants with low frequency (rare non-synonymous variants (<=2)) in the ESP is also shown. Click here for file
