# Supplementary material for: New population-based exome data question the pathogenicity of some genetic variants previously associated with Marfan syndrome
Source: BMC Genet. 2014 Jun 18;15:74. doi: 10.1186/1471-2156-15-74 (PMC4070351; doi:10.1186/1471-2156-15-74)
Supplement: Additional file 1 — The methods of prediction and genotyping a variant in Northern European control population. [file 1471-2156-15-74-S1.docx]

**Additional file 1: The methods of prediction**

By using *SIFT* predictions (version 5.1.0)^1^, variants were classified as “tolerant” (benign) or “damaging”. By *Polyphen-2* (version 2.2.2)^2^, variants were predicted to be “probably damaging”, “possibly damaging”, or “benign”. “Probably damaging” and “possibly damaging” were considered “damaging” in this study. *Grantham physicochemical score*^3^ was calculated using the Grantham amino acid difference matrix. In our study, the scores above 100 were considered radical (damaging), and the scores below 100 were considered conservative (benign)^4^. The degree of *conservation across species* were obtained from HGMD and searched in the website of [www.uniprot.org](http://www.uniprot.org/). Variants were classified as occurring at a position with no substitutions (conserved/damaging) or ≥1 substitution (not conserved/benign). Finally, each missense variants were also evaluated whether they were predicted to be damaging by ≥3 of the four applied prediction tools. A variant was classified as “damaging” if it has the agreement of ≥3 damaging predictions^4^.

**The method of genotyping a variant in Northern European control population**

In order to test if the ESP data harbored an overrepresentation of variants, using a Taqman assay, one of variant *TGFBR2* V387M (rs35766612) was genotyped in our own healthy control population of Northern European (n=750) as described previously^5^. This variant previously reported to involve in thoracic aortic aneurysms and dissections^6^ was presented in the ESP with 30 carriers. In brief, the DNA was extracted using the QIAamp DNA Blood Mini and Maxi kits (Qiagen, Hilden, Germany). SNP genotypes for rs35766612 was determined using fluorescence-based real-time polymerase chain reaction (PCR) (ABI PRISM 7900 Sequence Detection System; Applied Biosystems, Foster City, CA) and a predeveloped TaqMan assay (Applied Biosystems). An allelic discrimination run was performed allowing for discrimination between the allele compositions of each sample. The Context Sequence is GGACCTCAAGAGCTCCAATATCCTC[*A/G*]TGAAGAACGACCTAACCTGCTG. This control population consisted of men and women between the age of 55-75 years (mean age 63 years) with no history of MFS or other cardiac diseases. Their height was normal and none of them had any clinical manifestations of MFS.

References:

1. SIFT - Tool to predict nonsynonmous / missense variants [Internet]. Retrieved from: http://sift.bii.a-star.edu.sg

2. PolyPhen-2: prediction of functional effects of human nsSNPs [Internet]. Retrieved from: http://genetics.bwh.harvard.edu/pph2

3. Grantham R. Amino acid difference formula to help explain protein evolution. Science 1974,185(4154):862–864.

4. Giudicessi JR, Kapplinger JD, Tester DJ, Alders M, Salisbury BA, Wilde AAM, Ackerman MJ: Phylogenetic and physicochemical analyses enhance the classification of rare nonsynonymous single nucleotide variants in type 1 and 2 long-QT syndrome. Circ Cardiovasc Genet 2012, 5(5):519–528.

5. Olesen MS, Jespersen T, Nielsen JB, Liang B, Møller DV, Hedley P, Christiansen M, Varró A, Olesen SP, Haunsø S, Schmitt N, Svendsen JH: Mutations in sodium channel β-subunit SCN3B are associated with early-onset lone atrial fibrillation. Cardiovasc Res 2011, 89(4):786-793.

6. Mátyás G, Arnold E, Carrel T, Baumgartner D, Boileau C, Berger W, Steinmann B:Identification and in silico analyses of novel TGFBR1 and TGFBR2 mutations in Marfansyndrome-related disorders. Hum Mutat 2006, 27(8):760-769.
