# Supplementary material for: New population-based exome data question the pathogenicity of some genetic variants previously associated with Marfan syndrome
Source: BMC Genet. 2014 Jun 18;15:74. doi: 10.1186/1471-2156-15-74 (PMC4070351; doi:10.1186/1471-2156-15-74)
Supplement: Additional file 2: Table S1 — Variants associated with Marfan syndrome not present in ESP. [file 1471-2156-15-74-S2.docx]

Additional file 2: Table S1: Variants associated with Marfan syndrome not present in ESP

| Gene | Variant | Amino acid | Type | dbSNP ID | PolyPhen-2 | Grantham value | SIFT | Conservation | Agreement≥3  （ | reference |
| --- | --- | --- | --- | --- | --- | --- | --- | --- | --- | --- |
| *FBN1* | c.1A>T | p.M1L | Missense |  | Benign | B(15) | Tolerant | YES | B | ((1) (2) |
|  | c.32T>G | p.L11R | Missense |  | Probably damaging | D(102) | Damaging | YES | D | (3) |
|  | c.115G>C | p.A39P | Missense |  | Benign | B(27) | Tolerant | YES | B | (4) |
|  | c.164G>A | p.G55E | Missense |  | Probably damaging | B(98) | Damaging | NO | B | (5) |
|  | c.169A>G | p.N57D | Missense |  | Probably damaging | B(23) | Tolerant | YES | B | (6) |
|  | c.175T>C | p.C59R | Missense |  | Probably damaging | D(180) | Damaging | YES | D | (7) |
|  | c.184C>T | p.R62C | Missense | rs25403 | Probably damaging | D(180) | Damaging | YES | D | (8) (9) |
|  | c.199T>C | p.C67R | Missense |  | Probably damaging | D(180) | Damaging | YES | D | (10) |
|  | c.202T>A | p.C68S | Missense |  | Probably damaging | D(112) | Tolerant | YES | D | (11) |
|  | c.211T>C | p.W71R | Missense |  | Probably damaging | D(101) | Damaging | YES | D | (12) |
|  | c.214A>T | p.K72* | Nonsense |  |  |  |  |  |  | (13) |
|  | c.229G>T | p.G77* | Nonsense |  |  |  |  |  |  | (14) |
|  | c.238T>C | p.C80R | Missense | rs111764111 | Probably damaging | D(180) | Damaging | YES | D | (11) |
|  | c.238T>G | p.C80G | Missense |  | Probably damaging | D(159) | Damaging | YES | D | (3) |
|  | c.239G>A | p.C80Y | Missense |  | Probably damaging | D(194) | Damaging | YES | D | (15) |
|  | c.266G>T | p.C89F | Missense | rs112660651 | Probably damaging | D(205) | Tolerant | YES | D | (16) |
|  | c.267T>G | p.C89W | Missense |  | Probably damaging | D(215) | Damaging | YES | D | (11) |
|  | c.266G>A | p.C89Y | Missense |  | Probably damaging | D(194) | Damaging | YES | D | (17) |
|  | c.299G>A | p.C100Y | Missense |  | Probably damaging | D(194) | Damaging | YES | D | (6) |
|  | c.301A>G | p.T101A | Missense |  | Probably damaging | B(58) | Tolerant | YES | B | (11) |
|  | c.331T>C | p.C111R | Missense |  | Probably damaging | D(180) | Damaging | YES | D | (18) |
|  | c.344C>G | p.S115C | Missense |  | Probably damaging | D(112) | Tolerant | YES | D | (19) |
| *FBN1* | c.364C>T | p.R122C | Missense | rs137854467 | Probably damaging | D(180) | Tolerant | YES | D | (20) |
|  | c.368G>A | p.C123Y | Missense |  | Probably damaging | D(194) | Damaging | YES | D | (21) |
|  | c.370A>G | p.M124V | Missense |  | Possibly damaging | B(21) | Tolerant | YES | B | (22) |
|  | c.380G>A | p.G127D | Missense |  | Probably damaging | B(94) | Tolerant | YES | B | (23) |
|  | c.385T>G | p.C129G | Missense |  | Probably damaging | D(159) | Tolerant | YES | D | (24) |
|  | c.386G>A | p.C129Y | Missense |  | Probably damaging | D(194) | Damaging | YES | D | (25) |
|  | c.400T>G | p.C134G | Missense |  | Probably damaging | D(159) | Damaging | YES | D | (12) |
|  | c.401G>C | p.C134S | Missense |  | Probably damaging | D(112) | Damaging | YES | D | (15) |
|  | c.406T>A | p.C136S | Missense |  | Probably damaging | D(112) | Damaging | YES | D | (4) |
|  | c.433T>C | p.C145R | Missense |  | Probably damaging | D(180) | Damaging | YES | D | (26) |
|  | c.434G>A | p.C145Y | Missense |  | Probably damaging | D(194) | Damaging | YES | D | (15) |
|  | c.442C>T | p.P148S | Missense |  | Probably damaging | B(74) | Tolerant | YES | B | (11) |
|  | c.461G>C | p.C154S | Missense |  | Benign | D(112) | Damaging | YES | D | (27) |
|  | c.462T>A | p.C154* | Nonsense |  |  |  |  |  |  | (28) |
|  | c.478T>C | p.C160R | Missense |  | Probably damaging | D(180) | Damaging | YES | D | (23) |
|  | c.478T>G | p.C160G | Missense |  | Probably damaging | D(159) | Damaging | YES | D | (11) |
|  | c.479G>A | p.C160Y | Missense |  | Probably damaging | D(194) | Damaging | YES | D | (11) |
|  | c.491A>G | p.N164S | Missense |  | Probably damaging | B(46) | Tolerant | YES | B | (29) |
|  | c.493C>T | p.R165* | Nonsense |  |  |  |  |  |  | (28) |
|  | c.497G>T | p.C166F | Missense |  | Probably damaging | D(205) | Damaging | YES | D | (25) |
|  | c.496T>A | p.C166S | Missense |  | Probably damaging | D(112) | Damaging | YES | D | (27) |
|  | c.497G>C | p.C166S | Missense |  | Probably damaging | D(112) | Damaging | YES | D | (30) |
|  | c.504C>G | p.C168W | Missense |  | Probably damaging | D(215) | Damaging | YES | D | (31) |
|  | c.510C>G | p.Y170* | Nonsense |  |  |  |  |  |  | (32) |
| *FBN1* | c.526C>T | p.Q176* | Nonsense |  |  |  |  |  |  | (33) |
|  | c.529T>C | p.C177R | Missense | rs363853 | Probably damaging | D(180) | Damaging | YES | D | (21) |
|  | c.529T>A | p.C177S | Missense |  | Probably damaging | D(112) | Tolerant | YES | D | (4) |
|  | c.530G>A | p.C177Y | Missense |  | Probably damaging | D(194) | Damaging | YES | D | (4) |
|  | c.532G>A | p.E178* | Nonsense |  |  |  |  |  |  | (34) |
|  | c.586C>T | p.Q196* | Nonsense |  |  |  |  |  |  | (35) |
|  | c.626G>A | p.C209Y | Missense |  | Probably damaging | D(194) | Damaging | YES | D | (1) |
|  | c.629G>A | p.C210Y | Missense |  | Probably damaging | D(194) | Damaging | YES | D | (17) |
|  | c.640G>C | p.G214R | Missense |  | Probably damaging | D(125) | Damaging | YES | D | (36) |
|  | 640 G>A | p.G214S | Missense | rs113111224 | Probably damaging | B(56) | Damaging | YES | D | (37) |
|  | c.643C>T | p.R215* | Nonsense |  |  |  |  |  |  | (38) |
|  | c.649T>G | p.W217G | Missense |  | Probably damaging | D(184) | Tolerant | YES | D | (39) |
|  | c.651G>A | p.W217* | Nonsense |  |  |  |  |  |  | (40) |
|  | c.664G>T | p.E222* | Nonsense |  |  |  |  |  |  | (11) |
|  | c.679C>T | p.Q227* | Nonsense |  |  |  |  |  |  | (11) |
|  | c.714T>C | p.N238N | Missense |  |  |  |  |  |  | (1) |
|  | c.718C>T | p.R240C | Missense | rs137854480 | Probably damaging | D(180) | Damaging | YES | D | (41) |
|  | c.719G>A | p.R240H | Missense |  | Probably damaging | B(29) | Tolerant | YES | B | (42) |
|  | c.772C>T | p.Q258* | Nonsense |  |  |  |  |  |  | (12) |
|  | c.799G>A | p.G267R | Missense |  | Probably damaging | D(125) | Tolerant | YES | D | (39) |
|  | c.813C>A | p.C271* | Nonsense |  |  |  |  |  |  | (43) |
|  | c.850C>T | p.Q284* | Nonsense |  |  |  |  |  |  | (23) |
|  | c.876C>A | p.C292* | Nonsense |  |  |  |  |  |  | (11) |
|  | c.945T>A | p.C315* | Nonsense |  |  |  |  |  |  | (44) |
| *FBN1* | c.1011C>A | p.Y337* | Nonsense |  |  |  |  |  |  | (45) |
|  | c.1035C>A | p.C345* | Nonsense |  |  |  |  |  |  | (11) |
|  | c.1042C>T | p.Q348* | Nonsense |  |  |  |  |  |  | (21) |
|  | c.1051C>T | p.Q351* | Nonsense |  |  |  |  |  |  | (23) |
|  | c.1076G>A | p.C359Y | Missense |  | Probably damaging | D(194) | Damaging | YES | D | (46) |
|  | c.1093T>C | p.C365R | Missense |  | Probably damaging | D(180) | Tolerant | YES | D | (16) |
|  | c.1098G>T | p.W366C | Missense |  | Probably damaging | D(215) | Tolerant | YES | D | (16) |
|  | c.1097G>A | p.W366* | Nonsense |  |  |  |  |  |  | (23) |
|  | c.1098G>A | p.W366* | Nonsense |  |  |  |  |  |  | (45) |
|  | c.1147G>C | p.E383Q | Missense |  | Probably damaging | B(29) | Tolerant | NO | B | (2) |
|  | c.1147G>A | p.E383K | Missense |  | Probably damaging | B(56) | Tolerant | NO | B | (2) |
|  | c.1285C>T | p.R429* | Nonsense |  |  |  |  |  |  | (47) |
|  | c.1302T>G | p.Y434* | Nonsense |  |  |  |  |  |  | (33) |
|  | c.1315C>G | p.R439G | Missense |  | Probably damaging | D(125) | Tolerant | YES | D | (21) |
|  | c.1318G>T | p.E440* | Nonsense |  |  |  |  |  |  | (11) |
|  | c.1361A>C | p.Q454P | Missense |  | Benign | B(76) | Tolerant | YES | B | (21) |
|  | c.1373A>C | p.Y458S | Missense |  | Benign | D(144) | Tolerant | NO | B | (11) |
|  | c.1379G>C | p.C460S | Missense |  | Probably damaging | D(112) | Damaging | YES | D | (11) |
|  | c.1379G>A | p.C460Y | Missense |  | Probably damaging | D(194) | Damaging | YES | D | (22) |
|  | c.1381C>T | p.Q461* | Nonsense |  |  |  |  |  |  | (11) |
|  | c.1416C>A | p.Y472* | Nonsense |  |  |  |  |  |  | (11) |
|  | c.1421G>T | p.C474F | Missense |  | Probably damaging | D(205) | Damaging | YES | D | (48) |
|  | c.1422T>G | p.C474W | Missense |  | Probably damaging | D(215) | Damaging | YES | D | (49) |
|  | c.1426T>C | p.C476R | Missense |  | Probably damaging | D(180) | Damaging | YES | D | (22) |
| *FBN1* | c.1426T>G | p.C476G | Missense |  | Probably damaging | D(159) | Damaging | YES | D | (50) |
|  | c.1432A>T | p.K478* | Nonsense |  |  |  |  |  |  | (11) |
|  | c.1453C>T | p.R485C | Missense | rs137854485 | Probably damaging | D(180) | Tolerant | YES | D | (40) |
|  | c.1462T>C | p.C488R | Missense |  | Probably damaging | D(180) | Damaging | YES | D | (4) |
|  | c.1463G>T | p.C488F | Missense |  | Probably damaging | D(205) | Damaging | YES | D | (46) |
|  | c.1464T>A | p.C488* | Nonsense |  |  |  |  |  |  | (51) |
|  | c.1468G>T | p.D490Y | Missense |  | Probably damaging | D(160) | Tolerant | YES | D | (52) |
|  | c.1495T>C | p.C499R | Missense |  | Probably damaging | D(180) | Tolerant | YES | D | (53) |
|  | c.1495T>A | p.C499S | Missense |  | Probably damaging | D(112) | Damaging | YES | D | (54) |
|  | c.1496G>C | p.C499S | Missense |  | Probably damaging | D(112) | Damaging | YES | D | (5) |
|  | c.1496G>A | p.C499Y | Missense |  | Probably damaging | D(194) | Damaging | YES | D | (3) |
|  | c.1510T>C | p.C504R | Missense |  | Probably damaging | D(180) | Damaging | YES | D | (23) |
|  | c.1511G>T | p.C504F | Missense |  | Probably damaging | D(205) | Damaging | YES | D | (55) |
|  | c.1511G>A | p.C504Y | Missense |  | Probably damaging | D(194) | Damaging | YES | D | (15) |
|  | c.1546C>T | p.R516* | Nonsense |  |  |  |  |  |  | (21) |
|  | c.1556A>G | p.Y519C | Missense |  | Probably damaging | D(194) | Damaging | YES | D | (46) |
|  | c.1585C>T | p.R529* | Nonsense |  |  |  |  |  |  | (56) |
|  | c.1598A>G | p.E533G | Missense |  | Probably damaging | B(98) | Damaging | YES | D | (11) |
|  | c.1600T>C | p.C534R | Missense |  | Probably damaging | D(180) | Damaging | YES | D | (11) |
|  | c.1601G>A | p.C534Y | Missense |  | Probably damaging | D(194) | Damaging | YES | D | (57) |
|  | c.1606C>T | p.Q536* | Nonsense |  |  |  |  |  |  | (1) |
|  | c.1622G>A | p.C541Y | Missense |  | Probably damaging | D(194) | Damaging | YES | D | (28) |
|  | c.1630G>C | p.G544R | Missense |  | Probably damaging | D(125) | Damaging | YES | D | (11) |
|  | c.1633C>T | p.R545C | Missense |  | Probably damaging | D(180) | Damaging | YES | D | (18) |
| *FBN1* | c.1638C>G | p.C546W | Missense |  | Probably damaging | D(215) | Damaging | YES | D | (23) |
|  | c.1643A>T | p.N548I | Missense | rs137854462 | Probably damaging | D(149) | Damaging | YES | D | (58) |
|  | c.1678G>A | p.G560S | Missense |  | Probably damaging | B(56) | Damaging | YES | D | (16) |
|  | c.1708T>C | p.C570R | Missense | rs113902534 | Probably damaging | D(180) | Damaging | YES | D | (53) |
|  | c.1709G>C | p.C570S | Missense |  | Probably damaging | D(112) | Damaging | YES | D | (44) |
|  | c.1709G>A | p.C570Y | Missense |  | Probably damaging | D(194) | Damaging | YES | D | (16) |
|  | c.1711G>T | p.E571* | Nonsense |  |  |  |  |  |  | (8) |
|  | c.1721A>G | p.D574G | Missense |  | Probably damaging | B(94) | Damaging | YES | D | (45) |
|  | c.1727G>A | p.C576Y | Missense |  | Probably damaging | D(194) | Damaging | YES | D | (4) |
|  | c.1744T>C | p.C582R | Missense |  | Probably damaging | D(180) | Damaging | YES | D | (4) |
|  | c.1753G>C | p.G585R | Missense |  | Probably damaging | D(125) | Damaging | YES | D | (59) |
|  | c.1754G>A | p.G585E | Missense |  | Probably damaging | B(98) | Damaging | YES | D | (40) |
|  | c.1760G>A | p.C587Y | Missense |  | Probably damaging | D(194) | Damaging | YES | D | (60) |
|  | c.1775G>A | p.G592D | Missense |  | Probably damaging | B(94) | Damaging | YES | D | (16) |
|  | c.1786T>G | p.C596G | Missense |  | Probably damaging | D(159) | Damaging | YES | D | (44) |
|  | c.1787G>A | p.C596Y | Missense |  | Probably damaging | D(194) | Damaging | YES | D | (19) |
|  | c.1794C>G | p.C598W | Missense |  | Probably damaging | D(215) | Damaging | YES | D | (41) |
|  | c.1831T>C | p.C611R | Missense |  | Probably damaging | D(180) | Damaging | YES | D | (3) |
|  | c.1837G>A | p.D613N | Missense |  | Probably damaging | B(23) | Damaging | YES | D | (10) |
|  | c.1847A>G | p.E616G | Missense | rs25457 | Probably damaging | B(98) | Damaging | YES | D | (33) |
|  | c.1846G>A | p.E616K | Missense |  | Probably damaging | B(56) | Damaging | YES | D | (11) |
|  | c.1849T>G | p.C617G | Missense |  | Probably damaging | D(159) | Damaging | YES | D | (3) |
|  | c.1867T>G | p.C623G | Missense |  | Probably damaging | D(159) | Damaging | YES | D | (11) |
|  | c.1868G>T | p.C623F | Missense |  | Probably damaging | D(205) | Damaging | YES | D | (11) |
| *FBN1* | c.1879C>T | p.R627C | Missense |  | Probably damaging | D(180) | Tolerant | YES | D | (61) |
|  | c.1883G>A | p.C628Y | Missense |  | Probably damaging | D(194) | Damaging | YES | D | (26) |
|  | c.1890C>A | p.N630K | Missense |  | Probably damaging | B(94) | Damaging | YES | D | (11) |
|  | c.1900T>C | p.S634P | Missense |  | Probably damaging | B(74) | Damaging | YES | D | (29) |
|  | c.1904A>G | p.Y635C | Missense |  | Probably damaging | D(194) | Damaging | YES | D | (21) |
|  | c.1905C>G | p.Y635* | Nonsense |  |  |  |  |  |  | (14) |
|  | c.1907G>T | p.R636I | Missense |  | Benign | B(97) | Damaging | YES | B | (21) |
|  | c.1909T>C | p.C637R | Missense |  | Probably damaging | D(180) | Damaging | YES | D | (53) |
|  | c.1910G>T | p.C637F | Missense |  | Probably damaging | D(205) | Damaging | YES | D | (11) |
|  | c.1911T>G | p.C637W | Missense |  | Probably damaging | D(215) | Damaging | YES | D | (44) |
|  | Cys637Tyr | p.C637Y | Missense |  | Probably damaging | D(194) | Damaging | YES | D | (43) |
|  | c.1916G>A | p.C639Y | Missense |  | Probably damaging | D(194) | Damaging | YES | D | (15) |
|  | c.1928T>C | p.L643P | Missense |  | Probably damaging | B(98) | Damaging | NO | B | (11) |
|  | c.1948C>T | p.R650C | Missense |  | Probably damaging | D(180) | Damaging | YES | D | (22) |
|  | c.1955G>C | p.C652S | Missense |  | Probably damaging | D(112) | Damaging | YES | D | (27) |
|  | c.1955G>A | p.C652Y | Missense |  | Probably damaging | D(194) | Damaging | YES | D | (29) |
|  | c.1960G>A | p.D654N | Missense |  | Probably damaging | B(94) | Damaging | YES | D | (62) |
|  | c.1981T>C | p.C661R | Missense |  | Probably damaging | D(180) | Damaging | YES | D | (30) |
|  | c.1981T>G | p.C661G | Missense |  | Probably damaging | D(159) | Tolerant | YES | D | (11) |
|  | c.1982G>A | p.C661Y | Missense |  | Probably damaging | D(194) | Tolerant | YES | D | (19) |
|  | c.1995C>G | p.Y665* | Nonsense |  |  |  |  |  |  | (63) |
|  | c.2042C>A | p.S681Y | Missense |  | Probably damaging | D(144) | Damaging | YES | D | (19) |
|  | c.2047T>C | p.C683R | Missense |  | Probably damaging | D(180) | Damaging | YES | D | (19) |
|  | c.2048G>A | p.C683Y | Missense |  | Probably damaging | D(194) | Damaging | YES | D | (45) |
| *FBN1* | c.2051G>A | p.C684Y | Missense |  | Possibly damaging | D(194) | Damaging | YES | D | (4) |
|  | c.2055C>G | p.C685W | Missense |  | Probably damaging | D(215) | Damaging | YES | D | (19) |
|  | c.2054G>A | p.C685Y | Missense |  | Possibly damaging | D(194) | Damaging | YES | D | (3) |
|  | c.2057C>A | p.A686D | Missense |  | Probably damaging | D(126) | Damaging | YES | D | (11) |
|  | c.2097T>A | p.C699* | Nonsense |  |  |  |  |  |  | (21) |
|  | c.2113G>A | p.A705T | Missense |  | Possibly damaging | B(58) | Tolerant | NO | B | (64) |
|  | c.2122C>T | p.Q708* | Nonsense |  |  |  |  |  |  | (14) |
|  | c.2132G>A | p.C711Y | Missense |  | Probably damaging | D(194) | Damaging | YES | D | (64) |
|  | c.2161G>T | p.G721C | Missense |  | Probably damaging | D(159) | Damaging | YES | D | (4) |
|  | c.2168A>C | p.D723A | Missense | rs137854463 | Probably damaging | D(126) | Damaging | YES | D | (58) |
|  | c.2168A>T | p.D723V | Missense |  | Probably damaging | D(152) | Damaging | YES | D | (19) |
|  | c.2171T>G | p.I724R | Missense |  | Probably damaging | B(97) | Damaging | YES | D | (44) |
|  | c.2170A>G | p.I724V | Missense | rs113512280 | Possibly damaging | B(29) | Damaging | YES | D | (17) |
|  | c.2177A>G | p.E726G | Missense |  | Probably damaging | B(98) | Damaging | YES | D | (11) |
|  | c.2180G>A | p.C727Y | Missense |  | Probably damaging | D(194) | Damaging | YES | D | (19) |
|  | c.2201G>T | p.C734F | Missense |  | Probably damaging | D(205) | Damaging | YES | D | (19) |
|  | c.2215T>C | p.C739R | Missense |  | Probably damaging | D(180) | Damaging | YES | D | (11) |
|  | c.2237A>G | p.Y746C | Missense |  | Probably damaging | D(194) | Damaging | YES | D | (25) |
|  | c.2243G>A | p.C748Y | Missense |  | Probably damaging | D(194) | Damaging | YES | D | (62) |
|  | c.2248T>G | p.C750G | Missense |  | Probably damaging | D(159) | Damaging | YES | D | (65) |
|  | c.2248T>A | p.C750S | Missense |  | Probably damaging | D(112) | Damaging | YES | D | (11) |
|  | c.2250C>A | p.C750* | Nonsense |  |  |  |  |  |  | (28) |
|  | c.2255C>A | p.S752* | Nonsense |  |  |  |  |  |  | (23) |
|  | c.2261A>G | p.Y754C | Missense | rs137854479 | Probably damaging | D(194) | Damaging | NO | D | (66) |
| *FBN1* | c.2305T>C | p.C769R | Missense |  | Probably damaging | D(180) | Damaging | YES | D | (15) |
|  | c.2305T>A | p.C769S | Missense |  | Probably damaging | D(112) | Damaging | YES | D | (17) |
|  | c.2306G>A | p.C769Y | Missense |  | Probably damaging | D(194) | Damaging | YES | D | (11) |
|  | c.2326T>G | p.C776G | Missense |  | Probably damaging | D(159) | Damaging | YES | D | (19) |
|  | c.2327G>T | p.C776F | Missense |  | Probably damaging | D(205) | Damaging | YES | D | (11) |
|  | c.2328T>G | p.C776W | Missense |  | Probably damaging | D(215) | Damaging | YES | D | (26) |
|  | c.2327G>A | p.C776Y | Missense |  | Probably damaging | D(194) | Damaging | YES | D | (16) |
|  | c.2341T>C | p.C781R | Missense |  | Probably damaging | D(180) | Damaging | YES | D | (16) |
|  | c.2342G>A | p.C781Y | Missense |  | Probably damaging | D(194) | Damaging | YES | D | (28) |
|  | c.2368T>G | p.C790G | Missense |  | Probably damaging | D(159) | Damaging | YES | D | (11) |
|  | c.2369G>T | p.C790F | Missense |  | Probably damaging | D(205) | Damaging | YES | D | (11) |
|  | c.2368T>A | p.C790S | Missense |  | Probably damaging | D(112) | Damaging | YES | D | (67) |
|  | c.2369G>A | p.C790Y | Missense |  | Probably damaging | D(194) | Damaging | YES | D | (3) |
|  | c.2376C>A | p.C792* | Nonsense |  |  |  |  |  |  | (21) |
|  | c.2415T>G | p.C805W | Missense |  | Probably damaging | D(215) | Damaging | YES | D | (15) |
|  | c.2428G>T | p.E810* | Nonsense |  |  |  |  |  |  | (21) |
|  | c.2433C>A | p.C811* | Nonsense |  |  |  |  |  |  | (10) |
|  | c.2433C>G | p.C811W | Missense |  | Probably damaging | D(215) | Damaging | YES | D | (46) |
|  | c.2432G>A | p.C811Y | Missense |  | Probably damaging | D(194) | Damaging | YES | D | (3) |
|  | c.2438C>G | p.S813* | Nonsense |  |  |  |  |  |  | (38) |
|  | c.2446T>C | p.C816R | Missense |  | Probably damaging | D(180) | Damaging | YES | D | (4) |
|  | c.2446T>G | p.C816G | Missense |  | Probably damaging | D(159) | Damaging | YES | D | (68) |
|  | c.2447G>T | p.C816F | Missense |  | Probably damaging | D(205) | Damaging | YES | D | (17) |
|  | c.2447G>C | p.C816S | Missense |  | Probably damaging | D(112) | Damaging | YES | D | (41) |
| *FBN1* | c.2448C>A | p.C816* | Nonsense |  |  |  |  |  |  | (11) |
|  | c.2456G>A | p.G819E | Missense |  | Probably damaging | B(98) | Damaging | YES | D | (59) |
|  | c.2463C>A | p.C821* | Nonsense |  |  |  |  |  |  | (11) |
|  | c.2462G>A | p.C821Y | Missense |  | Probably damaging | D(194) | Damaging | YES | D | (10) |
|  | c.2473C>T | p.P825S | Missense |  | Benign | B(74) | Tolerant | NO | B | (15) |
|  | c.2483T>G | p.F828C | Missense |  | Probably damaging | D(205) | Damaging | YES | D | (23) |
|  | c.2488T>A | p.C830S | Missense |  | Probably damaging | D(112) | Damaging | YES | D | (11) |
|  | c.2489G>A | p.C830Y | Missense |  | Probably damaging | D(194) | Damaging | YES | D | (69) |
|  | c.2495G>T | p.C832F | Missense |  | Probably damaging | D(205) | Damaging | YES | D | (10) |
|  | c.2495G>A | p.C832Y | Missense |  | Probably damaging | D(194) | Damaging | YES | D | (33) |
|  | c.2513T>C | p.L838S | Missense |  | Probably damaging | D(145) | Damaging | YES | D | (11) |
|  | c.2557T>A | p.C853S | Missense |  | Probably damaging | D(112) | Damaging | YES | D | (3) |
|  | c.2559C>A | p.C853* | Nonsense |  |  |  |  |  |  | (15) |
|  | c.2562G>A | p.W854* | Nonsense |  |  |  |  |  |  | (46) |
|  | c.2563C>T | p.Q855* | Nonsense |  |  |  |  |  |  | (11) |
|  | c.2581C>T | p.R861* | Nonsense |  |  |  |  |  |  | (33) |
|  | c.2584T>C | p.C862R | Missense |  | Probably damaging | D(180) | Damaging | YES | D | (70) |
|  | c.2623T>C | p.C875R | Missense |  | Probably damaging | D(180) | Damaging | YES | D | (11) |
|  | c.2638G>A | p.G880S | Missense |  | Probably damaging | B(56) | Damaging | YES | D | (71) |
|  | c.2645C>T | p.A882V | Missense |  | Probably damaging | B(64) | Damaging | YES | D | (46) |
|  | c.2651G>A | p.G884E | Missense |  | Probably damaging | B(98） | Damaging | NO | B | (4) |
|  | c.2668T>C | p.C890R | Missense |  | Probably damaging | D(180) | Damaging | YES | D | (72) |
|  | c.2668T>G | p.C890G | Missense |  | Probably damaging | D(159) | Damaging | YES | D | (21) |
|  | c.2687G>A | p.C896Y | Missense |  | Probably damaging | D(194) | Damaging | YES | D | (11) |
| *FBN1* | c.2722T>C | p.C908R | Missense |  | Probably damaging | D(180) | Damaging | YES | D | (19) |
|  | c.2723G>A | p.C908Y | Missense |  | Probably damaging | D(194) | Damaging | YES | D | (73) |
|  | c.2728G>A | p.D910N | Missense |  | Probably damaging | B(23) | Tolerant | YES | B | (74) |
|  | c.2728G>C | p.D910H | Missense |  | Probably damaging | B(81) | Damaging | YES | D | (6) |
|  | c.2738A>G | p.E913G | Missense |  | Probably damaging | B(98) | Damaging | YES | D | (16) |
|  | c.2740T>A | p.C914S | Missense |  | Probably damaging | D(112) | Damaging | YES | D | (11) |
|  | c.2761T>G | p.C921G | Missense |  | Probably damaging | D(159) | Damaging | YES | D | (19) |
|  | c.2763T>A | p.C921* | Nonsense |  |  |  |  |  |  | (6) |
|  | c.2776T>C | p.C926R | Missense |  | Probably damaging | D(180) | Damaging | YES | D | (25) |
|  | c.2776T>G | p.C926G | Missense |  | Probably damaging | D(159) | Damaging | YES | D | (11) |
|  | c.2777G>A | p.C926Y | Missense |  | Probably damaging | D(194) | Damaging | YES | D | (69) |
|  | c.2785A>C | p.T929P | Missense |  | Possibly damaging | B(38) | Damaging | NO | D | (10) |
|  | c.2848T>C | p.C950R | Missense |  | Probably damaging | D(180) | Damaging | YES | D | (17) |
|  | c.2860C>T | p.R954C | Missense |  | Probably damaging | D(180) | Damaging | YES | D | (23) |
|  | c.2861G>A | p.R954H | Missense | rs112911555 | Probably damaging | B(29) | Damaging | YES | D | (2) |
|  | c.2896G>T | p.E966* | Nonsense |  |  |  |  |  |  | (23) |
|  | c.2920C>T | p.R974C | Missense |  | Probably damaging | D(180) | Damaging | YES | D | (23) |
|  | c.2938T>A | p.C980S | Missense |  | Probably damaging | D(112) | Damaging | YES | D | (45) |
|  | c.2939G>A | p.C980Y | Missense |  | Probably damaging | D(194) | Damaging | YES | D | (11) |
|  | c.2942G>C | p.C981S | Missense |  | Probably damaging | D(112) | Damaging | YES | D | (12) |
|  | c.2953G>A | p.G985R | Missense |  | Probably damaging | D(125) | Damaging | YES | D | (16) |
|  | c.2954G>A | p.G985E | Missense | rs137854477 | Probably damaging | B(98) | Damaging | YES | D | (75) |
|  | c.2980G>T | p.E994* | Nonsense |  |  |  |  |  |  | (76) |
|  | c.2986T>C | p.C996R | Missense | rs140592 | Probably damaging | D(180) | Damaging | YES | D | (41) |
| *FBN1* | c.2992A>G | p.M998V | Missense |  | Benign | B(21) | Tolerant | NO | B | (77) |
|  | c.3022T>C | p.C1008R | Missense |  | Probably damaging | D(180) | Damaging | YES | D | (78) |
|  | c.3023G>A | p.C1008Y | Missense |  | Probably damaging | D(194) | Damaging | YES | D | (4) |
|  | c.3037G>A | p.G1013R | Missense | rs140593 | Probably damaging | D(125) | Damaging | YES | D | (25) |
|  | c.3037G>C | p.G1013R | Missense |  | Probably damaging | D(125) | Damaging | YES | D | (41) |
|  | c.3043G>C | p.A1015P | Missense |  | Possibly damaging | B(27) | Tolerant | NO | B | (79) |
|  | c.3043G>A | p.A1015T | Missense | rs55831697 | Benign | B(58) | Tolerant | NO | B | (12) |
|  | c.3069G>C | p.K1023N | Missense |  | Possibly damaging | B(94) | Damaging | NO | B | (80) |
|  | c.3083A>G | p.D1028G | Missense |  | Probably damaging | B(94) | Damaging | YES | D | (23) |
|  | c.3082G>T | p.D1028Y | Missense |  | Probably damaging | D(160) | Damaging | YES | D | (81) |
|  | c.3083A>T | p.D1028V | Missense |  | Probably damaging | D(152) | Damaging | YES | D | (82) |
|  | c.3091G>T | p.E1031* | Nonsense |  |  |  |  |  |  | (10) |
|  | c.3095G>A | p.C1032Y | Missense | rs137854481 | Probably damaging | D(194) | Damaging | YES | D | (83) |
|  | c.3116G>A | p.C1039Y | Missense |  | Probably damaging | D(194) | Damaging | YES | D | (33) |
|  | c.3125G>A | p.G1042D | Missense |  | Probably damaging | B(94) | Damaging | YES | D | (12) |
|  | c.3124G>A | p.G1042S | Missense |  | Probably damaging | B(56) | Damaging | YES | D | (4) |
|  | c.3128A>G | p.K1043R | Missense | rs137854472 | Benign | B(26) | Tolerant | NO | B | (84) |
|  | c.3130T>C | p.C1044R | Missense |  | Probably damaging | D(180) | Damaging | YES | D | (85) |
|  | c.3132C>A | p.C1044* | Nonsense |  |  |  |  |  |  | (28) |
|  | c.3131G>A | p.C1044Y | Missense |  | Probably damaging | D(194) | Damaging | YES | D | (27) |
|  | c.3137A>G | p.N1046S | Missense |  | Probably damaging | B(46) | Damaging | YES | D | (45) |
|  | c.3143T>C | p.I1048T | Missense |  | Possibly damaging | B(89) | Damaging | YES | D | (86) |
|  | c.3157T>C | p.C1053R | Missense |  | Probably damaging | D(180) | Damaging | YES | D | (87) |
|  | c.3163T>G | p.C1055G | Missense |  | Probably damaging | D(159) | Damaging | YES | D | (64) |
| *FBN1* | c.3163T>A | p.C1055S | Missense |  | Probably damaging | D(112) | Damaging | YES | D | (40) |
|  | c.3165T>G | p.C1055W | Missense |  | Probably damaging | D(215) | Damaging | YES | D | (16) |
|  | c.3164G>A | p.C1055Y | Missense |  | Probably damaging | D(194) | Damaging | YES | D | (16) |
|  | c.3173G>A | p.G1058D | Missense |  | Probably damaging | B(94) | Damaging | YES | D | (21) |
|  | c.3188C>G | p.S1063C | Missense |  | Possibly damaging | D(112) | Tolerant | YES | D | (11) |
|  | c.3202T>G | p.C1068G | Missense |  | Probably damaging | D(159) | Damaging | YES | D | (85) |
|  | c.3209A>G | p.D1070G | Missense |  | Probably damaging | B(94) | Damaging | YES | D | (47) |
|  | c.3212T>G | p.I1071S | Missense |  | Probably damaging | D(142) | Damaging | YES | D | (88) |
|  | c.3215A>G | p.D1072G | Missense |  | Probably damaging | B(94) | Damaging | YES | D | (87) |
|  | c.3219A>T | p.E1073D | Missense |  | Probably damaging | B(45) | Damaging | YES | D | (88) |
|  | c.3217G>A | p.E1073K | Missense | rs137854478 | Probably damaging | B(56) | Damaging | YES | D | (25) |
|  | c.3220T>C | p.C1074R | Missense | rs137854465 | Probably damaging | D(180) | Damaging | YES | D | (80) |
|  | c.3229T>C | p.S1077P | Missense | rs140586 | Probably damaging | B(74) | Damaging | YES | D | (33) |
|  | c.3241T>G | p.C1081G | Missense |  | Probably damaging | D(159) | Damaging | YES | D | (47) |
|  | c.3256T>C | p.C1086R | Missense |  | Probably damaging | D(180) | Damaging | YES | D | (89) |
|  | c.3257G>A | p.C1086Y | Missense | rs137854484 | Probably damaging | D(194) | Damaging | YES | D | (53) |
|  | c.3263A>T | p.N1088I | Missense |  | Probably damaging | D(149) | Damaging | YES | D | (23) |
|  | c.3263A>G | p.N1088S | Missense |  | Probably damaging | B(46) | Damaging | YES | D | (44) |
|  | c.3268C>T | p.P1090S | Missense |  | Probably damaging | B(74) | Tolerant | YES | B | (3) |
|  | c.3285C>A | p.C1095* | Nonsense |  |  |  |  |  |  | (15) |
|  | c.3289T>C | p.C1097R | Missense |  | Probably damaging | D(180) | Damaging | YES | D | (85) |
|  | c.3295G>T | p.E1099* | Nonsense |  |  |  |  |  |  | (90) |
|  | c.3299G>T | p.G1100V | Missense |  | Probably damaging | D(109) | Damaging | YES | D | (46) |
|  | c.3302A>G | p.Y1101C | Missense |  | Probably damaging | D(194) | Damaging | YES | D | (16) |
| *FBN1* | c.3304G>T | p.E1102* | Nonsense |  |  |  |  |  |  | (45) |
|  | c.3332G>A | p.C1111Y | Missense |  | Probably damaging | D(194) | Damaging | YES | D | (26) |
|  | c.3338A>G | p.D1113G | Missense | rs140597 | Probably damaging | B(94) | Damaging | YES | D | (33) |
|  | c.3338A>T | p.D1113V | Missense |  | Probably damaging | D(152) | Damaging | YES | D | (28) |
|  | c.3344A>G | p.D1115G | Missense |  | Possibly damaging | B(94) | Damaging | YES | D | (91) |
|  | c.3349T>C | p.C1117R | Missense |  | Probably damaging | D(180) | Damaging | YES | D | (85) |
|  | c.3349T>G | p.C1117G | Missense |  | Probably damaging | D(159) | Damaging | YES | D | (87) |
|  | c.3350G>A | p.C1117Y | Missense | rs137854470 | Probably damaging | D(194) | Damaging | YES | D | (70) |
|  | c.3373C>T | p.R1125* | Nonsense |  |  |  |  |  |  | (28) |
|  | c.3386G>A | p.C1129Y | Missense | rs137854482 | Probably damaging | D(194) | Damaging | YES | D | (55) |
|  | c.3389A>C | p.H1130P | Missense |  | Benign | B(77) | Tolerant | NO | B | (6) |
|  | c.3393C>G | p.N1131K | Missense |  | Probably damaging | B(94) | Damaging | YES | D | (11) |
|  | c.3391A>T | p.N1131Y | Missense | rs137854473 | Probably damaging | D(143) | Damaging | YES | D | (84) |
|  | c.3408C>G | p.Y1136* | Nonsense |  |  |  |  |  |  | (4) |
|  | c.3410G>C | p.R1137P | Missense | rs137854456 | Possibly damaging | D(103) | Tolerant | YES | D | (92) |
|  | c.3412T>G | p.C1138G | Missense |  | Probably damaging | D(159) | Damaging | YES | D | (11) |
|  | c.3413G>C | p.C1138S | Missense |  | Probably damaging | D(112) | Damaging | YES | D | (11) |
|  | c.3413G>A | p.C1138Y | Missense |  | Probably damaging | D(194) | Damaging | YES | D | (23) |
|  | c.3419G>T | p.C1140F | Missense |  | Probably damaging | D(205) | Damaging | YES | D | (85) |
|  | c.3420C>A | p.C1140* | Nonsense |  |  |  |  |  |  | (23) |
|  | c.3455C>T | p.A1152V | Missense |  | Benign | B(64) | Tolerant | NO | B | (10) |
|  | c.3458G>C | p.C1153S | Missense |  | Probably damaging | D(112) | Damaging | YES | D | (21) |
|  | c.3458G>A | p.C1153Y | Missense | rs140599 | Probably damaging | D(194) | Damaging | YES | D | (64) |
|  | c.3463G>A | p.D1155N | Missense |  | Probably damaging | B(23) | Damaging | YES | D | (93) |
| *FBN1* | c.3464A>G | p.D1155G | Missense |  | Probably damaging | B(94) | Damaging | YES | D | (74) |
|  | c.3473A>G | p.E1158G | Missense |  | Probably damaging | B(98) | Damaging | YES | D | (23) |
|  | c.3497G>A | p.C1166Y | Missense |  | Probably damaging | D(194) | Damaging | YES | D | (94) |
|  | c.3503A>G | p.N1168S | Missense |  | Benign | B(46) | Tolerant | NO | B | (44) |
|  | c.3511T>C | p.C1171R | Missense |  | Probably damaging | D(180) | Damaging | YES | D | (53) |
|  | c.3513C>A | p.C1171* | Nonsense |  |  |  |  |  |  | (14) |
|  | c.3513C>G | p.C1171W | Missense |  | Probably damaging | D(215) | Damaging | YES | D | (18) |
|  | c.3519C>G | p.N1173K | Missense |  | Probably damaging | B(94) | Damaging | YES | D | (18) |
|  | c.3524T>C | p.I1175T | Missense |  | Benign | B(89) | Tolerant | YES | B | (41) |
|  | c.3526G>A | p.G1176R | Missense |  | Probably damaging | D(125) | Damaging | YES | D | (95) |
|  | c.3533A>G | p.Y1178C | Missense |  | Probably damaging | D(194) | Damaging | NO | D | (11) |
|  | c.3538T>C | p.C1180R | Missense |  | Probably damaging | D(180) | Damaging | YES | D | (11) |
|  | c.3545G>C | p.C1182S | Missense |  | Probably damaging | D(112) | Damaging | YES | D | (94) |
|  | c.3546C>G | p.C1182W | Missense |  | Probably damaging | D(215) | Damaging | YES | D | (96) |
|  | c.3554G>A | p.G1185D | Missense |  | Probably damaging | B(94) | Damaging | YES | D | (3) |
|  | c.3557A>G | p.Y1186C | Missense |  | Probably damaging | D(194) | Damaging | YES | D | (47) |
|  | c.3584G>A | p.C1195Y | Missense |  | Probably damaging | D(194) | Damaging | YES | D | (11) |
|  | c.3593T>C | p.I1198T | Missense |  | Benign | B(89) | Tolerant | YES | B | (11) |
|  | c.3596A>C | p.D1199A | Missense |  | Benign | D(126) | Damaging | YES | D | (5) |
|  | c.3599A>G | p.E1200G | Missense |  | Probably damaging | B(98) | Damaging | YES | D | (97) |
|  | c.3603C>A | p.C1201* | Nonsense |  |  |  |  |  |  | (44) |
|  | c.3602G>A | p.C1201Y | Missense |  | Probably damaging | D(194) | Damaging | YES | D | (98) |
|  | c.3656A>G | p.Y1219C | Missense |  | Probably damaging | D(194) | Damaging | YES | D | (21) |
|  | c.3667T>C | p.C1223R | Missense |  | Probably damaging | D(180) | Damaging | YES | D | (23) |
| *FBN1* | c.3668G>A | p.C1223Y | Missense | rs137854469 | Probably damaging | D(194) | Damaging | YES | D | (99) |
|  | c.3672G>T | p.Q1224H | Missense |  | Benign | B(24) | Tolerant | NO | B | (1) |
|  | c.3676G>T | p.G1226* | Nonsense |  |  |  |  |  |  | (79) |
|  | c.3703T>C | p.S1235P | Missense |  | Probably damaging | B(74) | Damaging | NO | B | (100) |
|  | c.3706T>C | p.C1236R | Missense |  | Probably damaging | D(180) | Damaging | YES | D | (101) |
|  | c.3712G>A | p.D1238N | Missense |  | Probably damaging | B(23) | Damaging | YES | D | (77) |
|  | c.3713A>G | p.D1238G | Missense |  | Probably damaging | B(94) | Damaging | YES | D | (91) |
|  | c.3722A>G | p.E1241G | Missense |  | Probably damaging | B(98) | Damaging | YES | D | (11) |
|  | c.3725G>A | p.C1242Y | Missense | rs137854471 | Probably damaging | D(194) | Damaging | YES | D | (80) |
|  | c.3745T>C | p.C1249R | Missense |  | Probably damaging | D(180) | Damaging | YES | D | (23) |
|  | c.3746G>C | p.C1249S | Missense | rs137854458 | Probably damaging | D(112) | Damaging | YES | D | (102) |
|  | c.3760T>A | p.C1254S | Missense |  | Probably damaging | D(112) | Damaging | YES | D | (103) |
|  | c.3778G>T | p.E1260* | Nonsense |  |  |  |  |  |  | (33) |
|  | c.3781T>G | p.Y1261D | Missense |  | Probably damaging | D(160) | Damaging | YES | D | (21) |
|  | c.3782A>G | p.Y1261C | Missense |  | Probably damaging | D(194) | Damaging | YES | D | (55) |
|  | c.3787T>C | p.C1263R | Missense |  | Probably damaging | D(180) | Damaging | YES | D | (2) |
|  | c.3793T>C | p.C1265R | Missense | rs137854474 | Probably damaging | D(180) | Damaging | YES | D | (104) |
|  | c.3793T>G | p.C1265G | Missense |  | Probably damaging | D(159) | Damaging | YES | D | (11) |
|  | c.3794G>A | p.C1265Y | Missense |  | Probably damaging | D(194) | Damaging | YES | D | (4) |
|  | c.3833G>C | p.C1278S | Missense |  | Probably damaging | D(112) | Damaging | YES | D | (21) |
|  | c.3850T>C | p.C1284R | Missense |  | Probably damaging | D(180) | Damaging | YES | D | (2) |
|  | c.3850T>G | p.C1284G | Missense |  | Probably damaging | D(159) | Damaging | YES | D | (28) |
|  | c.3851G>A | p.C1284Y | Missense |  | Probably damaging | D(194) | Damaging | YES | D | (3) |
|  | c.3898A>G | p.K1300E | Missense |  | Probably damaging | B(56) | Tolerant | YES | D | (91) |
| *FBN1* | c.3905C>A | p.S1302* | Nonsense |  |  |  |  |  |  | (11) |
|  | c.3920G>A | p.C1307Y | Missense |  | Probably damaging | D(194) | Damaging | YES | D | (23) |
|  | c.3929G>A | p.G1310D | Missense |  | Probably damaging | B(94) | Damaging | YES | D | (10) |
|  | c.3929G>T | p.G1310V | Missense |  | Probably damaging | D(109) | Damaging | YES | D | (15) |
|  | c.3958T>C | p.C1320R | Missense |  | Probably damaging | D(180) | Damaging | YES | D | (4) |
|  | c.3958T>A | p.C1320S | Missense | rs113393517 | Probably damaging | D(112) | Damaging | YES | D | (33) |
|  | c.3960T>A | p.C1320* | Nonsense |  |  |  |  |  |  | (21) |
|  | c.3965A>G | p.D1322G | Missense |  | Probably damaging | B(94) | Damaging | YES | D | (15) |
|  | c.3964G>C | p.D1322H | Missense |  | Probably damaging | B(81) | Damaging | YES | D | (74) |
|  | c.3973G>C | p.E1325Q | Missense |  | Probably damaging | B(29) | Damaging | YES | D | (27) |
|  | c.3973G>T | p.E1325* | Nonsense |  |  |  |  |  |  | (91) |
|  | c.3976T>C | p.C1326R | Missense |  | Probably damaging | D(180) | Damaging | YES | D | (53) |
|  | c.3997T>A | p.C1333S | Missense |  | Probably damaging | D(112) | Damaging | YES | D | (21) |
|  | c.4009G>C | p.A1337P | Missense |  | Probably damaging | B(27) | Damaging | YES | D | (41) |
|  | c.4016G>A | p.C1339Y | Missense |  | Probably damaging | D(194) | Damaging | YES | D | (16) |
|  | c.4022A>G | p.N1341S | Missense | rs140638 | Probably damaging | B(46) | Damaging | YES | D | (33) |
|  | c.4027G>A | p.A1343T | Missense |  | Possibly damaging | B(58) | Tolerant | YES | B | (11) |
|  | c.4030G>A | p.G1344R | Missense |  | Probably damaging | D(125) | Damaging | YES | D | (10) |
|  | c.4037T>G | p.F1346C | Missense |  | Probably damaging | D(205) | Damaging | YES | D | (79) |
|  | c.4038C>G | p.F1346L | Missense |  | Probably damaging | B(22) | Damaging | YES | D | (15) |
|  | c.4037T>C | p.F1346S | Missense |  | Probably damaging | D(155) | Damaging | YES | D | (23) |
|  | c.4044T>A | p.C1348* | Nonsense |  |  |  |  |  |  | (8) |
|  | c.4049G>T | p.C1350F | Missense |  | Probably damaging | D(205) | Damaging | YES | D | (3) |
|  | c.4049G>A | p.C1350Y | Missense |  | Probably damaging | D(194) | Damaging | YES | D | (11) |
| *FBN1* | c.4057G>A | p.G1353R | Missense |  | Probably damaging | D(125) | Damaging | YES | D | (11) |
|  | c.4082G>A | p.C1361Y | Missense |  | Probably damaging | D(194) | Damaging | YES | D | (53) |
|  | c.4096G>A | p.E1366K | Missense |  | Probably damaging | B(56) | Damaging | YES | D | (27) |
|  | c.4096G>T | p.E1366* | Nonsense |  |  |  |  |  |  | (35) |
|  | c.4099T>C | p.C1367R | Missense |  | Probably damaging | D(180) | Damaging | YES | D | (12) |
|  | c.4120T>G | p.C1374G | Missense |  | Probably damaging | D(159) | Damaging | YES | D | (1) |
|  | c.4121G>C | p.C1374S | Missense |  | Probably damaging | D(112) | Damaging | YES | D | (27) |
|  | c.4121G>A | p.C1374Y | Missense |  | Probably damaging | D(194) | Damaging | YES | D | (11) |
|  | c.4126C>T | p.Q1376* | Nonsense |  |  |  |  |  |  | (11) |
|  | c.4139G>A | p.C1380Y | Missense |  | Probably damaging | D(194) | Damaging | YES | D | (15) |
|  | c.4143G>C | p.K1381N | Missense |  | Benign | B(94) | Damaging | YES | B | (11) |
|  | c.4146T>A | p.N1382K | Missense |  | Probably damaging | B(94) | Damaging | YES | D | (15) |
|  | c.4145A>G | p.N1382S | Missense |  | Probably damaging | B(46) | Damaging | YES | D | (25) |
|  | c.4150A>T | p.M1384L | Missense |  | Benign | B(15) | Tolerant | YES | B | (5) |
|  | c.4160A>G | p.Y1387C | Missense |  | Possibly damaging | D(194) | Damaging | YES | D | (11) |
|  | c.4165T>C | p.C1389R | Missense |  | Probably damaging | D(180) | Damaging | YES | D | (27) |
|  | c.4172G>T | p.C1391F | Missense |  | Probably damaging | D(205) | Damaging | YES | D | (1) |
|  | c.4173C>A | p.C1391* | Nonsense |  |  |  |  |  |  | (21) |
|  | c.4192G>T | p.D1398N | Missense |  | Benign | B(23) | Damaging | YES | B | (5) |
|  | c.4204T>C | p.C1402R | Missense |  | Probably damaging | D(180) | Damaging | YES | D | (21) |
|  | c.4206T>G | p.C1402W | Missense |  | Probably damaging | D(215) | Damaging | YES | D | (53) |
|  | c.4205G>A | p.C1402Y | Missense |  | Probably damaging | D(194) | Damaging | YES | D | (49) |
|  | c.4210G>T | p.D1404Y | Missense |  | Probably damaging | D(160) | Damaging | YES | D | (18) |
|  | c.4214T>G | p.L1405R | Missense |  | Benign | D(102) | Tolerant | YES | B | (1) |
| *FBN1* | c.4217A>G | p.D1406G | Missense |  | Probably damaging | B(94) | Damaging | YES | D | (91) |
|  | c.4223G>T | p.C1408F | Missense |  | Probably damaging | D(205) | Damaging | YES | D | (91) |
|  | c.4259G>T | p.C1420F | Missense |  | Probably damaging | D(205) | Damaging | YES | D | (11) |
|  | c.4260C>G | p.C1420W | Missense |  | Probably damaging | D(215) | Damaging | YES | D | (45) |
|  | c.4261C>T | p.L1421F | Missense |  | Probably damaging | B(22) | Damaging | NO | D | (103) |
|  | c.4270C>T | p.P1424S | Missense |  | Possibly damaging | B(74) | Damaging | NO | B | (21) |
|  | c.4279T>G | p.Y1427D | Missense |  | Probably damaging | D(160) | Damaging | YES | D | (23) |
|  | c.4280A>G | p.Y1427C | Missense |  | Probably damaging | D(194) | Damaging | YES | D | (44) |
|  | c.4285T>A | p.C1429S | Missense |  | Probably damaging | D(112) | Damaging | YES | D | (41) |
|  | c.4293C>G | p.C1431W | Missense | rs112375043 | Probably damaging | D(215) | Damaging | YES | D | (3) |
|  | c.4292G>A | p.C1431Y | Missense |  | Probably damaging | D(194) | Damaging | YES | D | (3) |
|  | c.4348T>G | p.C1450G | Missense |  | Possibly damaging | D(159) | Damaging | YES | D | (17) |
|  | c.4349G>A | p.C1450Y | Missense |  | Probably damaging | D(194) | Damaging | YES | D | (2) |
|  | c.4367G>C | p.C1456S | Missense |  | Probably damaging | D(112) | Damaging | YES | D | (11) |
|  | c.4408T>C | p.C1470R | Missense |  | Probably damaging | D(180) | Damaging | YES | D | (15) |
|  | c.4409G>A | p.C1470Y | Missense |  | Probably damaging | D(194) | Damaging | YES | D | (47) |
|  | c.4423G>A | p.G1475S | Missense |  | Probably damaging | B(56) | Damaging | YES | D | (28) |
|  | c.4427A>G | p.Y1476C | Missense |  | Probably damaging | D(194) | Damaging | YES | D | (11) |
|  | c.4429G>T | p.E1477* | Nonsense |  |  |  |  |  |  | (33) |
|  | c.4453T>C | p.C1485R | Missense |  | Probably damaging | D(180) | Damaging | YES | D | (23) |
|  | c.4454G>A | p.C1485Y | Missense |  | Probably damaging | D(194) | Damaging | YES | D | (15) |
|  | c.4460A>C | p.D1487A | Missense |  | Probably damaging | D(126) | Damaging | YES | D | (3) |
|  | c.4467T>A | p.N1489K | Missense |  | Probably damaging | B(94) | Damaging | YES | D | (3) |
|  | c.4472G>T | p.C1491F | Missense |  | Probably damaging | D(205) | Damaging | YES | D | (79) |
| *FBN1* | c.4472G>A | p.C1491Y | Missense |  | Probably damaging | D(194) | Tolerant | YES | D | (2) |
|  | c.4490G>C | p.C1497S | Missense |  | Probably damaging | D(112) | Damaging | YES | D | (94) |
|  | c.4495A>T | p.S1499C | Missense |  | Probably damaging | D(112) | Damaging | NO | D | (12) |
|  | c.4505G>A | p.C1502Y | Missense |  | Probably damaging | D(194) | Damaging | YES | D | (69) |
|  | c.4537T>C | p.C1513R | Missense | rs112723282 | Probably damaging | D(180) | Damaging | YES | D | (80) |
|  | c.4539C>G | p.C1513W | Missense |  | Probably damaging | D(215) | Damaging | YES | D | (69) |
|  | c.4567C>T | p.R1523* | Nonsense |  |  |  |  |  |  | (105) |
|  | c.4577G>A | p.C1526Y | Missense |  | Probably damaging | D(194) | Damaging | YES | D | (10) |
|  | c.4582G>T | p.D1528Y | Missense |  | Probably damaging | D(160) | Damaging | YES | D | (23) |
|  | c.4602C>A | p.C1534* | Nonsense |  |  |  |  |  |  | (4) |
|  | c.4615C>T | p.R1539* | Nonsense |  |  |  |  |  |  | (91) |
|  | c.4621C>T | p.R1541* | Nonsense |  |  |  |  |  |  | (106) |
|  | c.4689C>G | p.C1563W | Missense |  | Probably damaging | D(215) | Damaging | YES | D | (11) |
|  | c.4691G>T | p.C1564F | Missense |  | Probably damaging | D(205) | Damaging | NO | D | (21) |
|  | c.4691G>A | p.C1564Y | Missense |  | Probably damaging | D(194) | Damaging | NO | D | (27) |
|  | c.4709G>A | p.W1570* | Nonsense |  |  |  |  |  |  | (44) |
|  | c.4727T>C | p.M1576T | Missense |  | Benign | B(81) | Tolerant | NO | B | (28) |
|  | c.4739T>G | p.V1580G | Missense |  | Benign | D(109) | Tolerant | YES | D | (80) |
|  | c.4750G>T | p.E1584* | Nonsense |  |  |  |  |  |  | (16) |
|  | c.4753T>A | p.Y1585N | Missense |  | Probably damaging | D(143) | Damaging | YES | D | (107) |
|  | c.4766G>T | p.C1589F | Missense |  | Probably damaging | D(205) | Damaging | YES | D | (70) |
|  | c.4777G>T | p.E1593* | Nonsense |  |  |  |  |  |  | (44) |
|  | c.4780G>A | p.G1594S | Missense |  | Probably damaging | B(56) | Damaging | YES | D | (26) |
|  | c.4781G>T | p.G1594V | Missense |  | Probably damaging | D(109) | Damaging | YES | D | (12) |
| *FBN1* | c.4786C>T | p.R1596* | Nonsense |  |  |  |  |  |  | (16) |
|  | c.4813G>A | p.E1605K | Missense |  | Probably damaging | B(56) | Damaging | YES | D | (33) |
|  | c.4828T>G | p.C1610G | Missense |  | Probably damaging | D(159) | Damaging | YES | D | (18) |
|  | c.4852C>T | p.Q1618* | Nonsense |  |  |  |  |  |  | (10) |
|  | c.4864T>C | p.C1622R | Missense |  | Probably damaging | D(180) | Damaging | YES | D | (23) |
|  | c.4888C>T | p.Q1630* | Nonsense |  |  |  |  |  |  | (11) |
|  | c.4891T>G | p.C1631G | Missense |  | Probably damaging | D(159) | Damaging | YES | D | (21) |
|  | c.4895G>A | p.R1632H | Missense |  | Benign | B(29) | Damaging | NO | B | (10) |
|  | c.4898G>C | p.C1633S | Missense |  | Probably damaging | D(112) | Damaging | YES | D | (23) |
|  | c.4925A>G | p.D1642G | Missense |  | Benign | B(94) | Tolerant | NO | B | (17) |
|  | c.4930C>T | p.R1644* | Nonsense |  |  |  |  |  |  | (16) |
|  | c.4955G>A | p.C1652Y | Missense |  | Probably damaging | D(194) | Damaging | YES | D | (38) |
|  | c.4973G>T | p.C1658F | Missense |  | Probably damaging | D(205) | Damaging | YES | D | (10) |
|  | c.4981G>A | p.G1661R | Missense |  | Probably damaging | D(125) | Damaging | YES | D | (59) |
|  | c.4987T>C | p.C1663R | Missense | rs137854459 | Probably damaging | D(180) | Damaging | YES | D | (108) |
|  | c.4988G>T | p.C1663F | Missense |  | Probably damaging | D(205) | Damaging | YES | D | (12) |
|  | c.4988G>A | p.C1663Y | Missense |  | Probably damaging | D(194) | Damaging | YES | D | (21) |
|  | c.4999G>A | p.V1667I | Missense | rs140626 | Benign | B(29) | Tolerant | NO | B | (33) |
|  | c.5014T>C | p.C1672R | Missense |  | Probably damaging | D(180) | Damaging | YES | D | (53) |
|  | c.5015G>T | p.C1672F | Missense | rs140627 | Probably damaging | D(205) | Damaging | YES | D | (53) |
|  | c.5015G>C | p.C1672S | Missense |  | Probably damaging | D(112) | Damaging | YES | D | (90) |
|  | c.5015G>A | p.C1672Y | Missense |  | Probably damaging | D(194) | Damaging | YES | D | (4) |
|  | c.5020T>G | p.C1674G | Missense |  | Probably damaging | D(159) | Damaging | YES | D | (4) |
|  | c.5021G>A | p.C1674Y | Missense |  | Probably damaging | D(194) | Damaging | YES | D | (15) |
| *FBN1* | c.5061C>A | p.C1687* | Nonsense |  |  |  |  |  |  | (3) |
|  | c.5156G>A | p.C1719Y | Missense |  | Probably damaging | D(194) | Damaging | YES | D | (11) |
|  | c.5159G>A | p.C1720Y | Missense |  | Probably damaging | D(194) | Damaging | YES | D | (23) |
|  | c.5162G>A | p.C1721Y | Missense |  | Probably damaging | D(194) | Damaging | YES | D | (41) |
|  | c.5182G>C | p.A1728P | Missense |  | Probably damaging | B(27) | Damaging | YES | D | (1) |
|  | c.5187G>A | p.W1729* | Nonsense |  |  |  |  |  |  | (28) |
|  | c.5203C>T | p.Q1735* | Nonsense |  |  |  |  |  |  | (4) |
|  | c.5296G>C | p.D1766H | Missense |  | Probably damaging | B(81) | Damaging | YES | D | (17) |
|  | c.5305G>A | p.E1769K | Missense |  | Probably damaging | B(56) | Damaging | YES | D | (47) |
|  | c.5309G>T | p.C1770F | Missense |  | Probably damaging | D(205) | Damaging | YES | D | (27) |
|  | c.5309G>A | p.C1770Y | Missense |  | Probably damaging | D(194) | Damaging | YES | D | (109) |
|  | c.5314G>T | p.E1772* | Nonsense |  |  |  |  |  |  | (15) |
|  | c.5330G>T | p.C1777F | Missense |  | Probably damaging | D(205) | Damaging | YES | D | (23) |
|  | c.5339G>A | p.G1780E | Missense |  | Probably damaging | B(98) | Damaging | YES | D | (59) |
|  | c.5342T>C | p.V1781A | Missense |  | Possibly damaging | B(64) | Tolerant | NO | B | (10) |
|  | c.5345G>A | p.C1782Y | Missense |  | Probably damaging | D(194) | Damaging | YES | D | (11) |
|  | c.5369G>C | p.R1790P | Missense |  | Possibly damaging | D(103) | Tolerant | YES | D | (16) |
|  | c.5368C>T | p.R1790* | Nonsense |  |  |  |  |  |  | (21) |
|  | c.5371T>C | p.C1791R | Missense |  | Probably damaging | D(180) | Damaging | YES | D | (28) |
|  | c.5372G>T | p.C1791F | Missense |  | Probably damaging | D(205) | Damaging | YES | D | (15) |
|  | c.5372G>A | p.C1791Y | Missense |  | Probably damaging | D(194) | Damaging | YES | D | (16) |
|  | c.5377T>C | p.C1793R | Missense |  | Probably damaging | D(180) | Damaging | YES | D | (15) |
|  | c.5379T>G | p.C1793W | Missense |  | Probably damaging | D(215) | Damaging | YES | D | (27) |
|  | c.5378G>A | p.C1793Y | Missense |  | Probably damaging | D(194) | Damaging | YES | D | (23) |
| *FBN1* | c.5386G>T | p.G1796* | Nonsense |  |  |  |  |  |  | (23) |
|  | c.5387G>T | p.G1796V | Missense |  | Probably damaging | D(109) | Damaging | YES | D | (23) |
|  | c.5404A>T | p.K1802* | Nonsense |  |  |  |  |  |  | (12) |
|  | c.5417G>C | p.C1806S | Missense |  | Probably damaging | D(112) | Damaging | YES | D | (19) |
|  | c.5417G>A | p.C1806Y | Missense |  | Probably damaging | D(194) | Damaging | YES | D | (71) |
|  | c.5431G>A | p.E1811K | Missense |  | Probably damaging | B(56) | Damaging | YES | D | (23) |
|  | c.5434T>C | p.C1812R | Missense |  | Probably damaging | D(180) | Damaging | YES | D | (6) |
|  | c.5435G>A | p.C1812Y | Missense |  | Probably damaging | D(194) | Damaging | YES | D | (23) |
|  | c.5452T>G | p.C1818G | Missense |  | Probably damaging | D(159) | Damaging | YES | D | (45) |
|  | c.5453G>A | p.C1818Y | Missense |  | Probably damaging | D(194) | Damaging | YES | D | (94) |
|  | c.5467G>T | p.E1823* | Nonsense |  |  |  |  |  |  | (94) |
|  | c.5471G>A | p.C1824Y | Missense |  | Probably damaging | D(194) | Damaging | YES | D | (11) |
|  | c.5477A>G | p.N1826S | Missense |  | Probably damaging | B(46) | Tolerant | YES | B | (11) |
|  | c.5479A>C | p.T1827P | Missense |  | Probably damaging | B(38) | Tolerant | YES | B | (45) |
|  | c.5488A>T | p.S1830C | Missense |  | Probably damaging | D(112) | Damaging | YES | D | (23) |
|  | c.5494C>T | p.R1832C | Missense |  | Probably damaging | D(180) | Damaging | YES | D | (41) |
|  | c.5497T>C | p.C1833R | Missense |  | Probably damaging | D(180) | Damaging | YES | D | (15) |
|  | c.5498G>C | p.C1833S | Missense |  | Probably damaging | D(112) | Damaging | YES | D | (55) |
|  | c.5504G>T | p.C1835F | Missense |  | Probably damaging | D(205) | Damaging | YES | D | (23) |
|  | c.5504G>A | p.C1835Y | Missense | rs111929350 | Probably damaging | D(194) | Damaging | YES | D | (106) |
|  | c.5509C>T | p.P1837S | Missense |  | Probably damaging | B(74) | Tolerant | YES | B | (93) |
|  | c.5512G>T | p.G1838C | Missense |  | Probably damaging | D(194) | Damaging | YES | D | (69) |
|  | c.5539T>C | p.C1847R | Missense |  | Probably damaging | D(180) | Damaging | YES | D | (12) |
|  | c.5541C>G | p.C1847W | Missense |  | Probably damaging | D(215) | Damaging | YES | D | (23) |
| *FBN1* | c.5560C>T | p.Q1854* | Nonsense |  |  |  |  |  |  | (11) |
|  | c.5578T>C | p.C1860R | Missense |  | Probably damaging | D(180) | Damaging | YES | D | (4) |
|  | c.5579G>T | p.C1860F | Missense |  | Probably damaging | D(205) | Damaging | YES | D | (11) |
|  | c.5579G>A | p.C1860Y | Missense |  | Probably damaging | D(194) | Damaging | YES | D | (4) |
|  | c.5593T>C | p.C1865R | Missense |  | Probably damaging | D(180) | Damaging | NO | D | (5) |
|  | c.5602A>C | p.T1868P | Missense |  | Probably damaging | B(38) | Tolerant | YES | B | (10) |
|  | c.5627G>A | p.C1876Y | Missense | rs112728248 | Probably damaging | D(194) | Damaging | YES | D | (21) |
|  | c.5636G>A | p.G1879D | Missense |  | Probably damaging | B(94) | Damaging | YES | D | (23) |
|  | c.5635G>T | p.G1879C | Missense |  | Probably damaging | D(159) | Damaging | YES | D | (11) |
|  | c.5660C>T | p.T1887I | Missense |  | Possibly damaging | B(89) | Damaging | NO | B | (21) |
|  | c.5666G>T | p.C1889F | Missense |  | Probably damaging | D(205) | Damaging | YES | D | (11) |
|  | c.5666G>C | p.C1889S | Missense |  | Probably damaging | D(112) | Damaging | YES | D | (45) |
|  | c.5671G>C | p.D1891H | Missense |  | Probably damaging | B(81) | Damaging | YES | D | (74) |
|  | c.5679T>A | p.N1893K | Missense |  | Possibly damaging | B(94) | Damaging | YES | D | (18) |
|  | c.5680G>A | p.E1894K | Missense |  | Probably damaging | B(56) | Damaging | YES | D | (4) |
|  | c.5683T>C | p.C1895R | Missense |  | Probably damaging | D(180) | Damaging | YES | D | (103) |
|  | c.5684G>A | p.C1895Y | Missense |  | Probably damaging | D(194) | Damaging | YES | D | (11) |
|  | c.5699G>T | p.C1900F | Missense |  | Probably damaging | D(205) | Damaging | YES | D | (10) |
|  | c.5699G>C | p.C1900S | Missense |  | Probably damaging | D(112) | Damaging | YES | D | (11) |
|  | c.5699G>A | p.C1900Y | Missense |  | Probably damaging | D(194) | Damaging | YES | D | (21) |
|  | c.5707G>A | p.G1903R | Missense |  | Probably damaging | D(125) | Damaging | YES | D | (26) |
|  | c.5713T>C | p.C1905R | Missense |  | Probably damaging | D(180) | Damaging | YES | D | (11) |
|  | c.5720A>G | p.N1907S | Missense |  | Probably damaging | B(46) | Damaging | YES | D | (23) |
|  | c.5723C>T | p.T1908I | Missense |  | Probably damaging | B(89) | Tolerant | YES | D | (71) |
| *FBN1* | c.5726T>G | p.I1909S | Missense |  | Benign | D(142) | Damaging | YES | D | (11) |
|  | c.5726T>C | p.I1909T | Missense |  | Benign | B(89) | Damaging | YES | B | (16) |
|  | c.5729G>T | p.G1910V | Missense |  | Probably damaging | D(109) | Damaging | YES | D | (94) |
|  | c.5743C>A | p.R1915S | Missense |  | Possibly damaging | D(110) | Tolerant | YES | D | (16) |
|  | c.5746T>A | p.C1916S | Missense |  | Probably damaging | D(112) | Damaging | YES | D | (11) |
|  | c.5756G>A | p.G1919D | Missense |  | Possibly damaging | B(94) | Damaging | YES | D | (71) |
|  | c.5756G>T | p.G1919V | Missense |  | Probably damaging | D(109) | Damaging | YES | D | (10) |
|  | c.5776A>G | p.N1926D | Missense |  | Possibly damaging | B(23) | Tolerant | NO | B | (79) |
|  | c.5782T>C | p.C1928R | Missense |  | Probably damaging | D(180) | Damaging | YES | D | (25) |
|  | c.5782T>G | p.C1928G | Missense |  | Probably damaging | D(159) | Damaging | YES | D | (28) |
|  | c.5783G>C | p.C1928S | Missense |  | Possibly damaging | D(112) | Damaging | YES | D | (42) |
|  | c.5783G>A | p.C1928Y | Missense |  | Probably damaging | D(194) | Damaging | YES | D | (28) |
|  | c.5788G>A | p.D1930N | Missense |  | Probably damaging | B(23) | Tolerant | YES | B | (33) |
|  | c.5789A>G | p.D1930G | Missense |  | Probably damaging | B(94) | Damaging | YES | D | (107) |
|  | c.5788G>C | p.D1930H | Missense |  | Probably damaging | B(81) | Damaging | YES | D | (23) |
|  | c.5798A>T | p.E1933V | Missense |  | Probably damaging | D(121) | Damaging | YES | D | (10) |
|  | c.5800T>G | p.C1934G | Missense |  | Probably damaging | D(159) | Damaging | YES | D | (4) |
|  | c.5800T>A | p.C1934S | Missense |  | Probably damaging | D(112) | Damaging | YES | D | (3) |
|  | c.5809G>T | p.G1937* | Nonsense |  |  |  |  |  |  | (46) |
|  | c.5836C>T | p.Q1946* | Nonsense |  |  |  |  |  |  | (21) |
|  | c.5861T>G | p.F1954C | Missense |  | Probably damaging | D(205) | Damaging | YES | D | (10) |
|  | c.5866T>C | p.C1956R | Missense |  | Probably damaging | D(180) | Damaging | YES | D | (17) |
|  | c.5873G>A | p.C1958Y | Missense |  | Probably damaging | D(194) | Damaging | YES | D | (44) |
|  | c.5912G>A | p.C1971Y | Missense |  | Probably damaging | D(194) | Tolerant | YES | D | (16) |
| *FBN1* | c.5927A>G | p.E1976G | Missense |  | Benign | B(98) | Damaging | YES | B | (3) |
|  | c.5926G>A | p.E1976K | Missense |  | Possibly damaging | B(56) | Tolerant | YES | B | (15) |
|  | c.5929T>C | p.C1977R | Missense |  | Probably damaging | D(180) | Damaging | YES | D | (62) |
|  | c.5931T>G | p.C1977W | Missense |  | Probably damaging | D(215) | Damaging | YES | D | (4) |
|  | c.5930G>A | p.C1977Y | Missense |  | Probably damaging | D(194) | Damaging | YES | D | (41) |
|  | c.5950T>C | p.C1984R | Missense |  | Probably damaging | D(180) | Damaging | YES | D | (3) |
|  | c.5959G>C | p.G1987R | Missense |  | Probably damaging | D(125) | Damaging | YES | D | (49) |
|  | c.5960G>T | p.G1987V | Missense |  | Possibly damaging | D(109) | Damaging | YES | D | (11) |
|  | c.5993G>A | p.C1998Y | Missense |  | Probably damaging | D(194) | Damaging | YES | D | (19) |
|  | c.5999G>C | p.C2000S | Missense |  | Possibly damaging | D(112) | Damaging | YES | D | (15) |
|  | c.6031T>G | p.C2011G | Missense |  | Probably damaging | D(159) | Damaging | YES | D | (15) |
|  | c.6037G>T | p.D2013Y | Missense |  | Probably damaging | D(160) | Damaging | YES | D | (74) |
|  | c.6046G>A | p.E2016K | Missense |  | Probably damaging | B(56) | Damaging | YES | D | (11) |
|  | c.6049T>C | p.C2017R | Missense |  | Probably damaging | D(180) | Damaging | YES | D | (33) |
|  | c.6068T>C | p.I2023T | Missense | rs363803 | Probably damaging | B(89) | Tolerant | NO | B | (33) |
|  | c.6087C>A | p.C2029* | Nonsense |  |  |  |  |  |  | (11) |
|  | c.6113G>T | p.C2038F | Missense |  | Probably damaging | D(205) | Damaging | YES | D | (11) |
|  | c.6113G>A | p.C2038Y | Missense | rs363804 | Probably damaging | D(194) | Damaging | YES | D | (28) |
|  | c.6115C>G | p.L2039V | Missense |  | Possibly damaging | B(32) | Tolerant | NO | B | (33) |
|  | c.6158G>T | p.C2053F | Missense | rs363805 | Benign | D(205) | Damaging | YES | D | (53) |
|  | c.6159C>A | p.C2053* | Nonsense |  |  |  |  |  |  | (23) |
|  | c.6161A>C | p.Q2054P | Missense |  | Possibly damaging | B(76) | Tolerant | YES | B | (38) |
|  | c.6160C>T | p.Q2054* | Nonsense |  |  |  |  |  |  | (16) |
|  | c.6170G>A | p.R2057Q | Missense | rs181032147 | Benign | B(43) | Damaging | YES | B | (33) |
| *FBN1* | c.6169C>T | p.R2057* | Nonsense |  |  |  |  |  |  | (33) |
|  | c.6181T>C | p.C2061R | Missense |  | Probably damaging | D(180) | Damaging | YES | D | (11) |
|  | c.6182G>T | p.C2061F | Missense |  | Probably damaging | D(194) | Damaging | YES | D | (110) |
|  | c.6186T>A | p.Y2062* | Nonsense |  |  |  |  |  |  | (4) |
|  | c.6190A>T | p.K2064* | Nonsense |  |  |  |  |  |  | (4) |
|  | c.6236C>G | p.S2079C | Missense |  | Probably damaging | D(112) | Damaging | YES | D | (12) |
|  | c.6241C>T | p.Q2081* | Nonsense |  |  |  |  |  |  | (45) |
|  | c.6248G>T | p.C2083F | Missense |  | Probably damaging | D(205) | Damaging | YES | D | (11) |
|  | c.6251G>C | p.C2084S | Missense |  | Benign | D(112) | Damaging | YES | D | (26) |
|  | c.6252C>G | p.C2084W | Missense |  | Probably damaging | D(215) | Damaging | YES | D | (6) |
|  | c.6251G>A | p.C2084Y | Missense |  | Probably damaging | D(194) | Tolerant | YES | D | (4) |
|  | c.6253T>C | p.C2085R | Missense |  | Probably damaging | D(180) | Damaging | YES | D | (28) |
|  | c.6296G>T | p.C2099F | Missense |  | Possibly damaging | D(205) | Damaging | YES | D | (44) |
|  | c.6297C>G | p.C2099W | Missense |  | Benign | D(215) | Damaging | YES | D | (18) |
|  | c.6296G>A | p.C2099Y | Missense |  | Possibly damaging | D(194) | Damaging | YES | D | (11) |
|  | c.6298C>T | p.P2100S | Missense |  | Probably damaging | B(74) | Tolerant | YES | B | (11) |
|  | c.6302C>A | p.T2101K | Missense |  | Benign | B(78) | Tolerant | NO | B | (47) |
|  | c.6313G>A | p.E2105K | Missense |  | Benign | B(56) | Tolerant | NO | B | (23) |
|  | c.6325C>T | p.Q2109* | Nonsense |  |  |  |  |  |  | (11) |
|  | c.6331T>C | p.C2111R | Missense | rs363815 | Possibly damaging | D(180) | Damaging | YES | D | (8) |
|  | c.6332G>A | p.C2111Y | Missense |  | Probably damaging | D(194) | Damaging | YES | D | (18) |
|  | c.6339T>A | p.Y2113* | Nonsense |  |  |  |  |  |  | (41) |
|  | c.6339T>G | p.Y2113* | Nonsense |  |  |  |  |  |  | (41) |
|  | c.6354C>G | p.I2118M | Missense |  | Possibly damaging | B(10) | Tolerant | NO | B | (23) |
| *FBN1* | c.6381T>A | p.D2127E | Missense |  | Possibly damaging | B(45) | Damaging | YES | D | (80) |
|  | c.6379G>T | p.D2127Y | Missense |  | Probably damaging | D(160) | Tolerant | YES | D | (38) |
|  | c.6380A>T | p.D2127V | Missense |  | Benign | D(152) | Damaging | YES | D | (81) |
|  | c.6388G>A | p.E2130K | Missense |  | Probably damaging | B(56) | Damaging | YES | D | (17) |
|  | c.6407T>A | p.V2136D | Missense |  | Benign | D(152) | Tolerant | NO | B | (23) |
|  | c.6418G>A | p.G2140R | Missense |  | Probably damaging | D(125) | Damaging | YES | D | (59) |
|  | c.6419G>A | p.G2140E | Missense |  | Probably damaging | B(98) | Damaging | YES | D | (59) |
|  | c.6425G>A | p.C2142Y | Missense |  | Probably damaging | D(194) | Damaging | YES | D | (55) |
|  | c.6430A>G | p.N2144D | Missense |  | Probably damaging | B(23) | Damaging | YES | D | (23) |
|  | c.6431A>G | p.N2144S | Missense | rs137854461 | Probably damaging | B(46) | Damaging | YES | D | (111) |
|  | c.6433A>C | p.T2145P | Missense |  | Probably damaging | B(38) | Damaging | YES | D | (23) |
|  | c.6451T>C | p.C2151R | Missense |  | Probably damaging | D(180) | Damaging | YES | D | (11) |
|  | c.6453C>G | p.C2151W | Missense |  | Probably damaging | D(215) | Damaging | YES | D | (80) |
|  | c.6458G>A | p.C2153Y | Missense |  | Probably damaging | D(194) | Damaging | YES | D | (49) |
|  | c.6478G>C | p.A2160P | Missense |  | Benign | B(27) | Tolerant | NO | B | (21) |
|  | c.6496G>A | p.D2166N | Missense |  | Possibly damaging | B(23) | Damaging | YES | D | (3) |
|  | c.6503A>G | p.D2168G | Missense |  | Possibly damaging | B(94) | Damaging | YES | D | (15) |
|  | c.6505G>T | p.E2169* | Nonsense |  |  |  |  |  |  | (23) |
|  | c.6509G>T | p.C2170F | Missense | rs363821 | Probably damaging | D(205) | Damaging | YES | D | (112) |
|  | c.6508T>A | p.C2170S | Missense |  | Probably damaging | D(112) | Damaging | YES | D | (17) |
|  | c.6517G>A | p.G2173S | Missense |  | Probably damaging | B(56) | Tolerant | YES | B | (44) |
|  | c.6554T>C | p.I2185T | Missense |  | Possibly damaging | B(89) | Tolerant | NO | B | (23) |
|  | c.6577G>T | p.E2193* | Nonsense |  |  |  |  |  |  | (27) |
|  | c.6580G>T | p.E2194* | Nonsense |  |  |  |  |  |  | (113) |
| *FBN1* | c.6583G>A | p.G2195R | Missense |  | Probably damaging | D(125) | Damaging | YES | D | (23) |
|  | c.6583G>T | p.G2195* | Nonsense |  |  |  |  |  |  | (16) |
|  | c.6658C>T | p.R2220* | Nonsense |  |  |  |  |  |  | (38) |
|  | c.6661T>C | p.C2221R | Missense | rs113543334 | Probably damaging | D(180) | Damaging | YES | D | (53) |
|  | c.6661T>G | p.C2221G | Missense |  | Probably damaging | D(159) | Damaging | YES | D | (114) |
|  | c.6662G>T | p.C2221F | Missense |  | Probably damaging | D(205) | Damaging | YES | D | (21) |
|  | c.6662G>C | p.C2221S | Missense | rs137854460 | Probably damaging | D(112) | Damaging | YES | D | (108) |
|  | c.6667A>C | p.N2223H | Missense |  | Probably damaging | B(68) | Damaging | YES | D | (16) |
|  | c.6670A>C | p.T2224P | Missense |  | Probably damaging | B(38) | Damaging | YES | D | (23) |
|  | c.6685G>T | p.E2229* | Nonsense |  |  |  |  |  |  | (23) |
|  | c.6694T>C | p.C2232R | Missense |  | Probably damaging | D(180) | Damaging | YES | D | (26) |
|  | c.6695G>A | p.C2232Y | Missense |  | Probably damaging | D(194) | Damaging | YES | D | (96) |
|  | c.6740A>G | p.D2247G | Missense |  | Probably damaging | B(94) | Damaging | YES | D | (11) |
|  | c.6739G>T | p.D2247Y | Missense |  | Probably damaging | D(160) | Damaging | YES | D | (17) |
|  | c.6740A>T | p.D2247V | Missense |  | Probably damaging | D(152) | Damaging | YES | D | (45) |
|  | c.6751T>C | p.C2251R | Missense | rs112836174 | Probably damaging | D(180) | Damaging | YES | D | (71) |
|  | c.6751T>A | p.C2251S | Missense |  | Probably damaging | D(112) | Damaging | YES | D | (77) |
|  | c.6772T>C | p.C2258R | Missense |  | Probably damaging | D(180) | Damaging | YES | D | (18) |
|  | c.6773G>A | p.C2258Y | Missense |  | Probably damaging | D(194) | Damaging | YES | D | (106) |
|  | c.6784C>T | p.Q2262* | Nonsense |  |  |  |  |  |  | (25) |
|  | c.6794G>T | p.C2265F | Missense |  | Probably damaging | D(205) | Damaging | YES | D | (11) |
|  | c.6794G>A | p.C2265Y | Missense |  | Probably damaging | D(194) | Damaging | YES | D | (40) |
|  | c.6800A>T | p.N2267I | Missense |  | Probably damaging | D(149) | Damaging | YES | D | (11) |
|  | c.6806T>C | p.I2269T | Missense |  | Possibly damaging | B(89) | Damaging | YES | D | (33) |
| *FBN1* | c.6809G>A | p.G2270D | Missense |  | Probably damaging | B(94) | Damaging | YES | D | (15) |
|  | c.6818T>C | p.M2273T | Missense |  | Benign | B(81) | Tolerant | NO | B | (23) |
|  | c.6822C>G | p.C2274W | Missense |  | Probably damaging | D(215) | Damaging | YES | D | (23) |
|  | c.6827G>A | p.C2276Y | Missense |  | Probably damaging | D(194) | Damaging | YES | D | (36) |
|  | c.6844C>T | p.R2282W | Missense |  | Probably damaging | D(101) | Tolerant | YES | D | (18) |
|  | c.6850C>A | p.P2284T | Missense |  | Possibly damaging | B(38) | Tolerant | YES | B | (4) |
|  | c.6867T>G | p.C2289W | Missense |  | Probably damaging | D(215) | Damaging | YES | D | (23) |
|  | c.6866G>A | p.C2289Y | Missense |  | Probably damaging | D(194) | Tolerant | YES | D | (11) |
|  | c.6871G>A | p.D2291N | Missense |  | Probably damaging | B(23) | Damaging | YES | D | (81) |
|  | c.6881A>C | p.E2294A | Missense |  | Probably damaging | D(107) | Damaging | YES | D | (46) |
|  | c.6883T>C | p.C2295R | Missense |  | Probably damaging | D(180) | Damaging | YES | D | (36) |
|  | c.6884G>A | p.C2295Y | Missense |  | Probably damaging | D(194) | Damaging | YES | D | (47) |
|  | c.6892A>T | p.K2298* | Nonsense |  |  |  |  |  |  | (6) |
|  | c.6906T>A | p.C2302* | Nonsense |  |  |  |  |  |  | (79) |
|  | c.6905G>A | p.C2302Y | Missense |  | Probably damaging | D(194) | Tolerant | YES | D | (23) |
|  | c.6919T>C | p.C2307R | Missense |  | Probably damaging | D(180) | Damaging | YES | D | (11) |
|  | c.6920G>C | p.C2307S | Missense | rs137854457 | Probably damaging | D(112) | Damaging | YES | D | (102) |
|  | c.6946T>C | p.C2316R | Missense |  | Probably damaging | D(180) | Damaging | YES | D | (11) |
|  | c.6952T>C | p.C2318R | Missense | rs111588631 | Probably damaging | D(180) | Damaging | YES | D | (11) |
|  | c.6953G>A | p.C2318Y | Missense |  | Probably damaging | D(194) | Damaging | YES | D | (11) |
|  | c.6993C>A | p.C2331* | Nonsense |  |  |  |  |  |  | (1) |
|  | c.7003C>T | p.R2335W | Missense |  | Probably damaging | D(101) | Damaging | YES | D | (19) |
|  | c.7015T>C | p.C2339R | Missense |  | Probably damaging | D(180) | Damaging | YES | D | (1) |
|  | c.7015T>G | p.C2339G | Missense |  | Probably damaging | D(159) | Damaging | YES | D | (44) |
| *FBN1* | c.7016G>A | p.C2339Y | Missense |  | Probably damaging | D(194) | Damaging | YES | D | (19) |
|  | c.7094G>A | p.C2365Y | Missense |  | Probably damaging | D(194) | Damaging | YES | D | (17) |
|  | c.7111T>C | p.W2371R | Missense |  | Probably damaging | D(101) | Damaging | YES | D | (10) |
|  | c.7112G>A | p.W2371* | Nonsense |  |  |  |  |  |  | (3) |
|  | c.7141C>T | p.Q2381* | Nonsense |  |  |  |  |  |  | (11) |
|  | c.7153G>A | p.A2385T | Missense |  | Possibly damaging | B(58) | Tolerant | YES | B | (21) |
|  | c.7168T>A | p.C2390S | Missense |  | Possibly damaging | D(112) | Damaging | YES | D | (56) |
|  | c.7180C>T | p.R2394* | Nonsense |  |  |  |  |  |  | (106) |
|  | c.7217G>A | p.C2406Y | Missense |  | Probably damaging | D(194) | Damaging | YES | D | (16) |
|  | c.7237T>C | p.C2413R | Missense |  | Possibly damaging | D(180) | Damaging | YES | D | (11) |
|  | c.7240C>T | p.R2414* | Nonsense |  |  |  |  |  |  | (8) |
|  | c.7275T>A | p.Y2425* | Nonsense |  |  |  |  |  |  | (11) |
|  | c.7285T>C | p.C2429R | Missense |  | Probably damaging | D(180) | Damaging | YES | D | (11) |
|  | c.7324T>G | p.C2442G | Missense |  | Possibly damaging | D(159) | Damaging | YES | D | (11) |
|  | c.7324T>A | p.C2442S | Missense |  | Possibly damaging | D(112) | Damaging | YES | D | (3) |
|  | c.7326T>A | p.C2442* | Nonsense |  |  |  |  |  |  | (11) |
|  | c.7326T>G | p.C2442W | Missense |  | Probably damaging | D(215) | Damaging | YES | D | (27) |
|  | c.7331A>G | p.D2444G | Missense |  | Probably damaging | B(94) | Damaging | YES | D | (74) |
|  | c.7339G>A | p.E2447K | Missense | rs137854464 | Probably damaging | B(56) | Damaging | YES | D | (80) |
|  | c.7339G>T | p.E2447* | Nonsense |  |  |  |  |  |  | (21) |
|  | c.7342T>C | p.C2448R | Missense |  | Probably damaging | D(180) | Damaging | YES | D | (12) |
|  | c.7364G>C | p.C2455S | Missense |  | Probably damaging | D(112) | Damaging | YES | D | (2) |
|  | c.7364G>A | p.C2455Y | Missense |  | Probably damaging | D(194) | Damaging | YES | D | (15) |
|  | c.7382A>C | p.N2461T | Missense |  | Benign | B(65) | Damaging | YES | B | (11) |
| *FBN1* | c.7398C>A | p.Y2466* | Nonsense |  |  |  |  |  |  | (62) |
|  | c.7399C>T | p.Q2467* | Nonsense |  |  |  |  |  |  | (62) |
|  | c.7410C>A | p.C2470* | Nonsense |  |  |  |  |  |  | (12) |
|  | c.7410C>G | p.C2470W | Missense |  | Probably damaging | D(215) | Damaging | YES | D | (23) |
|  | c.7409G>A | p.C2470Y | Missense |  | Probably damaging | D(194) | Damaging | YES | D | (4) |
|  | c.7429C>T | p.Q2477* | Nonsense |  |  |  |  |  |  | (11) |
|  | c.7448G>A | p.C2483Y | Missense |  | Probably damaging | D(194) | Damaging | YES | D | (73) |
|  | c.7454A>T | p.D2485V | Missense |  | Possibly damaging | D(152) | Damaging | YES | D | (115) |
|  | c.7465T>C | p.C2489R | Missense |  | Probably damaging | D(180) | Damaging | YES | D | (18) |
|  | c.7465T>G | p.C2489G | Missense |  | Probably damaging | D(159) | Damaging | YES | D | (116) |
|  | c.7466G>A | p.C2489Y | Missense |  | Probably damaging | D(194) | Damaging | YES | D | (95) |
|  | c.7487G>A | p.C2496Y | Missense |  | Probably damaging | D(194) | Damaging | YES | D | (10) |
|  | c.7498T>C | p.C2500R | Missense |  | Probably damaging | D(180) | Damaging | YES | D | (21) |
|  | c.7498T>A | p.C2500S | Missense | rs363810 | Probably damaging | D(112) | Damaging | YES | D | (112) |
|  | c.7499G>A | p.C2500Y | Missense |  | Probably damaging | D(194) | Damaging | YES | D | (21) |
|  | c.7526G>A | p.C2509Y | Missense |  | Probably damaging | D(194) | Damaging | YES | D | (11) |
|  | c.7531T>C | p.C2511R | Missense |  | Probably damaging | D(180) | Damaging | YES | D | (80) |
|  | c.7532G>A | p.C2511Y | Missense |  | Probably damaging | D(194) | Damaging | YES | D | (77) |
|  | c.7540G>A | p.G2514R | Missense | rs363811 | Probably damaging | D(125) | Damaging | YES | D | (112) |
|  | c.7540G>T | p.G2514* | Nonsense |  |  |  |  |  |  | (15) |
|  | c.7547C>T | p.T2516I | Missense |  | Probably damaging | B(89) | Damaging | YES | D | (23) |
|  | c.7559C>T | p.T2520M | Missense |  | Probably damaging | B(81) | Tolerant | NO | B | (23) |
|  | c.7565G>C | p.C2522S | Missense |  | Probably damaging | D(112) | Damaging | YES | D | (117) |
|  | c.7565G>A | p.C2522Y | Missense |  | Probably damaging | D(194) | Damaging | YES | D | (6) |
| *FBN1* | c.7577A>G | p.N2526S | Missense |  | Probably damaging | B(46) | Tolerant | YES | B | (23) |
|  | c.7605C>A | p.C2535* | Nonsense |  |  |  |  |  |  | (117) |
|  | c.7605C>G | p.C2535W | Missense | rs113544411 | Probably damaging | D(215) | Damaging | YES | D | (21) |
|  | c.7606G>A | p.G2536R | Missense |  | Probably damaging | D(125) | Tolerant | YES | D | (49) |
|  | c.7622G>T | p.C2541F | Missense |  | Probably damaging | D(205) | Damaging | YES | D | (23) |
|  | c.7624C>T | p.Q2542* | Nonsense |  |  |  |  |  |  | (23) |
|  | c.7633C>T | p.P2545S | Missense |  | Probably damaging | B(74) | Tolerant | YES | B | (26) |
|  | c.7657C>T | p.Q2553* | Nonsense |  |  |  |  |  |  | (118) |
|  | c.7664G>T | p.G2555V | Missense |  | Probably damaging | D(109) | Damaging | YES | D | (23) |
|  | c.7670C>A | p.S2557* | Nonsense |  |  |  |  |  |  | (15) |
|  | c.7681A>C | p.T2561P | Missense |  | Benign | B(38) | Tolerant | NO | B | (4) |
|  | c.7708G>A | p.E2570K | Missense |  | Probably damaging | B(56) | Damaging | YES | D | (21) |
|  | c.7711T>C | p.C2571R | Missense |  | Probably damaging | D(180) | Damaging | YES | D | (21) |
|  | c.7713T>A | p.C2571* | Nonsense |  |  |  |  |  |  | (23) |
|  | c.7726C>T | p.R2576C | Missense | rs147195031 | Probably damaging | D(180) | Tolerant | YES | D | (119) |
|  | c.7729T>C | p.C2577R | Missense |  | Probably damaging | D(180) | Damaging | YES | D | (96) |
|  | c.7730G>A | p.C2577Y | Missense |  | Probably damaging | D(194) | Damaging | YES | D | (23) |
|  | c.7742G>T | p.C2581F | Missense |  | Probably damaging | D(205) | Damaging | YES | D | (16) |
|  | c.7741T>A | p.C2581S | Missense |  | Probably damaging | D(112) | Damaging | YES | D | (115) |
|  | c.7743C>G | p.C2581W | Missense |  | Probably damaging | D(215) | Damaging | YES | D | (8) |
|  | c.7742G>A | p.C2581Y | Missense |  | Probably damaging | D(194) | Damaging | YES | D | (15) |
|  | c.7754T>C | p.I2585T | Missense |  | Possibly damaging | B(89) | Tolerant | NO | B | (33) |
|  | c.7756G>T | p.G2586W | Missense |  | Probably damaging | D(184) | Damaging | YES | D | (11) |
|  | c.7769G>A | p.C2590Y | Missense |  | Probably damaging | D(194) | Damaging | YES | D | (11) |
| *FBN1* | c.7774T>A | p.C2592S | Missense |  | Possibly damaging | D(112) | Damaging | YES | D | (21) |
|  | c.7783G>A | p.G2595S | Missense |  | Probably damaging | B(56) | Damaging | YES | D | (11) |
|  | c.7800C>A | p.Y2600* | Nonsense |  |  |  |  |  |  | (14) |
|  | c.7801C>T | p.Q2601* | Nonsense |  |  |  |  |  |  | (40) |
|  | c.7806G>A | p.W2602* | Nonsense |  |  |  |  |  |  | (11) |
|  | c.7815T>A | p.C2605* | Nonsense |  |  |  |  |  |  | (2) |
|  | c.7814G>A | p.C2605Y | Missense |  | Probably damaging | D(194) | Damaging | YES | D | (28) |
|  | c.7819G>A | p.D2607N | Missense |  | Probably damaging | B(23) | Damaging | YES | D | (74) |
|  | c.7820A>G | p.D2607G | Missense |  | Probably damaging | B(94) | Damaging | YES | D | (33) |
|  | c.7828G>A | p.E2610K | Missense | rs111984349 | Probably damaging | B(56) | Damaging | YES | D | (33) |
|  | c.7851C>G | p.C2617W | Missense |  | Probably damaging | D(215) | Damaging | YES | D | (11) |
|  | c.7864T>A | p.C2622S | Missense |  | Probably damaging | D(112) | Damaging | YES | D | (46) |
|  | c.7868A>C | p.H2623P | Missense |  | Probably damaging | B(77) | Tolerant | YES | B | (30) |
|  | c.7872C>G | p.N2624K | Missense |  | Probably damaging | B(94) | Damaging | YES | D | (16) |
|  | c.7871A>G | p.N2624S | Missense |  | Probably damaging | B(46) | Tolerant | YES | B | (117) |
|  | c.7871A>C | p.N2624T | Missense |  | Probably damaging | B(65) | Damaging | YES | D | (11) |
|  | c.7879G>A | p.G2627R | Missense |  | Probably damaging | D(125) | Damaging | YES | D | (102) |
|  | c.7886A>G | p.Y2629C | Missense |  | Probably damaging | D(194) | Damaging | NO | D | (21) |
|  | c.7915T>A | p.Y2639N | Missense |  | Possibly damaging | D(143) | Damaging | YES | D | (47) |
|  | c.7916A>G | p.Y2639C | Missense |  | Possibly damaging | D(194) | Damaging | YES | D | (47) |
|  | c.7936T>C | p.C2646R | Missense |  | Probably damaging | D(180) | Damaging | YES | D | (3) |
|  | c.7954T>G | p.C2652G | Missense |  | Probably damaging | D(159) | Damaging | YES | D | (8) |
|  | c.7955G>A | p.C2652Y | Missense |  | Probably damaging | D(194) | Damaging | YES | D | (15) |
|  | c.7966C>T | p.Q2656* | Nonsense |  |  |  |  |  |  | (11) |
| *FBN1* | c.7977C>A | p.C2659* | Nonsense |  |  |  |  |  |  | (14) |
|  | c.7978A>C | p.S2660R | Missense |  | Benign | D(110) | Tolerant | NO | B | (12) |
|  | c.7988G>C | p.C2663S | Missense |  | Probably damaging | D(112) | Damaging | YES | D | (35) |
|  | c.8003G>A | p.G2668D | Missense |  | Probably damaging | B(94) | Damaging | YES | D | (47) |
|  | c.8002G>T | p.G2668C | Missense |  | Probably damaging | D(159) | Damaging | YES | D | (16) |
|  | c.8006G>T | p.G2669V | Missense |  | Possibly damaging | D(109) | Damaging | YES | D | (11) |
|  | c.8056T>C | p.C2686R | Missense |  | Probably damaging | D(180) | Tolerant | YES | D | (15) |
|  | c.8057G>T | p.C2686F | Missense |  | Probably damaging | D(205) | Damaging | YES | D | (33) |
|  | c.8080C>T | p.R2694* | Nonsense |  |  |  |  |  |  | (27) |
|  | c.8123A>G | p.N2708S | Missense |  | Possibly damaging | B(46) | Tolerant | YES | B | (6) |
|  | c.8149G>T | p.E2717* | Nonsense |  |  |  |  |  |  | (16) |
|  | c.8224G>T | p.E2742* | Nonsense |  |  |  |  |  |  | (15) |
|  | c.8268G>A | p.W2756* | Nonsense |  |  |  |  |  |  | (120) |
|  | c.8320A>T | p.K2774* | Nonsense |  |  |  |  |  |  | (4) |
|  | c.8326C>T | p.R2776* | Nonsense |  |  |  |  |  |  | (121) |
|  | c.8339T>C | p.L2780P | Missense |  | Probably damaging | B(98) | Damaging | NO | B | (62) |
|  | c.8378A>G | p.Y2793C | Missense |  | Probably damaging | D(194) | Damaging | YES | D | (17) |
|  | c.8377T>C | p.Y2793H | Missense | rs113722038 | Probably damaging | B(83) | Damaging | YES | D | (3) |
|  | c.8379C>G | p.Y2793* | Nonsense |  |  |  |  |  |  | (21) |
|  | c.8422C>T | p.Q2808* | Nonsense |  |  |  |  |  |  | (17) |
|  | c.8518A>T | p.K2840* | Nonsense |  |  |  |  |  |  | (23) |
|  | c.8521G>T | p.E2841* | Nonsense |  |  |  |  |  |  | (44) |
|  | c.8547T>G | p.Y2849* | Nonsense |  |  |  |  |  |  | (122) |
|  | c.8599C>T | p.Q2867* | Nonsense |  |  |  |  |  |  | (11) |
| *TGFBR2* | c.287C>T | p.T96I | missense |  | Probably Damaging | B(89) | Tolerantd | YES | B | (123) |
|  | c.569G>A | p.R190H | missense |  | Probably Damaging | B(29) | Tolerant | NO | B | (124) |
|  | c.740A>T | p.D247V | missense |  | Probably Damaging | D(152) | Damaging | YES | D | (124) |
|  | c.761G>A | p.R254H | missense |  | Probably Damaging | B(29) | Damaging | YES | D | (123) |
|  | c.973A>C | p.T325P | missense |  | Probably Damaging | B(38) | Damaging | NO | B | (124) |
|  | c.1069G>A | p.G357R | missense |  | Probably Damaging | D(125) | Damaging | YES | D | (124) |
|  | c.923T>C | p.L308P | missense | rs28934568 | Probably Damaging | B(98) | Damaging | YES | D | (125) |
|  | c.1132A>G | p.R378G | missense |  | Probably Damaging | D(112) | Damaging | YES | D | (123) |
|  | c.1151A>G | p.N384S | missense |  | Probably Damaging | B(46) | Damaging | YES | D | (126) |
|  | c.1188T>G | p.C396W | missense |  | Probably Damaging | D(215) | Damaging | YES | D | (126) |
|  | c.1273A>G | p. M425V | missense | rs104893817 | Probably Damaging | B(21) | Damaging | YES | D | (127) |
|  | c.1276G>A | p.A426T | missense |  | Probably Damaging | B(58) | Damaging | NO | B | (123) |
| *TGFBR2* | c.1322C>T | p.S441F | missense |  | Probably Damaging | D(155) | Damaging | YES | D | (126) |
|  | c.1336G>A | p.D446N | missense |  | Probably Damaging | B(23) | Damaging | YES | D | (127) |
|  | c.1346C>T | p.S449F | missense | rs104893807 | Probably Damaging | D(155) | Damaging | YES | D | (125) |
|  | c.1358T>A | p.V453E | missense |  | Probably Damaging | D(121) | Damaging | YES | D | (128) |
|  | c.1484G>A | p.R495Q | missense |  | Probably Damaging | B(43) | Damaging | YES | D | (129) |
|  | c.1489C>T | p.R497* | Nonsense |  |  |  |  |  |  | (126) |
|  | c.1492C>A | p.P498T | missense |  | Probably Damaging | B(38) | Damaging | YES | D | (129) |
|  | c.1511G>A | p.W504* | Nonsense |  |  |  |  |  |  | (123) |
|  | c.1531C>T | p.Q511* | Nonsense |  |  |  |  |  |  | (123) |
|  | c.1561T>C | p.W521R | missense |  | Probably Damaging | D(101) | Damaging | YES | D | (130) |
|  | c.1562G>A | p.W521* | Nonsense |  |  |  |  |  |  | (123) |
|  | c.1574C>G | p.P525R | missense |  | Probably Damaging | D(103) | Damaging | YES | D | (123) |
|  | c.1589C>T | p.T530I | missense |  | Probably Damaging | B(89) | Damaging | NO | B | (124) |
|  | c.1609C>T | p.R537C | missense | rs104893809 | Probably Damaging | D(180) | Damaging | NO | D | (125) |
| *TGFBR1* | c.759G>A | p.M253I | missense |  | Benign | B(10) | Damaging | YES | B | (131) |
|  | c.799A>C | p.N267H | missense |  | Probably Damaging | B(68) | Damaging | YES | D | (132) |
|  | c.934G>A | p.G312S | missense |  | Probably Damaging | B(56) | Damaging | YES | D | (131) |
|  | c.1003A>C | p.K335Q | missense |  | Probably Damaging | B(53) | Damaging | YES | D | (133) |

Polyphen-2 predictions “Probably damaging” and “Possibly damaging” were considered “damaging” (pathogenic).

Grantham values ≥100 were defined as radical changes (pathogenic), and values < 100 as conservative (Benign).

The degree of conservation across species was obtained from HGMD and all variants with ≥1 substitution were classified as not conserved/(Benign).

Variants are classified as pathogenic if ≥3 of the in silico prediction tools predicted pathogenicity or Benign if <3 predicted pathogenicity.

References:

1. Rybczynski M, Bernhardt AMJ, Rehder U, Fuisting B, Meiss L, Voss U, et al. The spectrum of syndromes and manifestations in individuals screened for suspected Marfan syndrome. Am J Med Genet A. 2008 Dec 15;146A(24):3157–66.

2. Söylen B, Singh KK, Abuzainin A, Rommel K, Becker H, Arslan-Kirchner M, et al. Prevalence of dural ectasia in 63 gene-mutation-positive patients with features of Marfan syndrome type 1 and Loeys-Dietz syndrome and report of 22 novel FBN1 mutations. Clin Genet. 2009 Mar;75(3):265–70.

3. Baetens M, Van Laer L, De Leeneer K, Hellemans J, De Schrijver J, Van De Voorde H, et al. Applying massive parallel sequencing to molecular diagnosis of Marfan and Loeys-Dietz syndromes. Hum Mutat. 2011 Sep;32(9):1053–62.

4. Attanasio M, Lapini I, Evangelisti L, Lucarini L, Giusti B, Porciani M, et al. FBN1 mutation screening of patients with Marfan syndrome and related disorders: detection of 46 novel FBN1 mutations. Clin Genet. 2008 Jul;74(1):39–46.

5. Wang W-J, Han P, Zheng J, Hu F-Y, Zhu Y, Xie J-S, et al. Exon 47 skipping of fibrillin-1 leads preferentially to cardiovascular defects in patients with thoracic aortic aneurysms and dissections. J Mol Med Berl Ger. 2013 Jan;91(1):37–47.

6. Chung BH-Y, Lam ST-S, Tong TM-F, Li SY-H, Lun K-S, Chan DH-C, et al. Identification of novel FBN1 and TGFBR2 mutations in 65 probands with Marfan syndrome or Marfan-like phenotypes. Am J Med Genet A. 2009 Jul;149A(7):1452–9.

7. Wei X, Ju X, Yi X, Zhu Q, Qu N, Liu T, et al. Identification of sequence variants in genetic disease-causing genes using targeted next-generation sequencing. PloS One. 2011;6(12):e29500.

8. Körkkö J, Kaitila I, Lönnqvist L, Peltonen L, Ala-Kokko L. Sensitivity of conformation sensitive gel electrophoresis in detecting mutations in Marfan syndrome and related conditions. J Med Genet. 2002 Jan;39(1):34–41.

9. Yu R, Lai Z, Zhou W, Ti D-D, Zhang X-N. Recurrent FBN1 mutation (R62C) in a Chinese family with isolated ectopia lentis. Am J Ophthalmol. 2006 Jun;141(6):1136–8.

10. Hung C-C, Lin S-Y, Lee C-N, Cheng H-Y, Lin S-P, Chen M-R, et al. Mutation spectrum of the fibrillin-1 (FBN1) gene in Taiwanese patients with Marfan syndrome. Ann Hum Genet. 2009 Nov;73(Pt 6):559–67.

11. Stheneur C, Collod-Béroud G, Faivre L, Buyck JF, Gouya L, Le Parc J-M, et al. Identification of the minimal combination of clinical features in probands for efficient mutation detection in the FBN1 gene. Eur J Hum Genet EJHG. 2009 Sep;17(9):1121–8.

12. Sakai H, Visser R, Ikegawa S, Ito E, Numabe H, Watanabe Y, et al. Comprehensive genetic analysis of relevant four genes in 49 patients with Marfan syndrome or Marfan-related phenotypes. Am J Med Genet A. 2006 Aug 15;140(16):1719–25.

13. Kuchtey J, Chang TC, Panagis L, Kuchtey RW. Marfan syndrome caused by a novel FBN1 mutation with associated pigmentary glaucoma. Am J Med Genet A. 2013 Apr;161A(4):880–3.

14. Magyar I, Colman D, Arnold E, Baumgartner D, Bottani A, Fokstuen S, et al. Quantitative sequence analysis of FBN1 premature termination codons provides evidence for incomplete NMD in leukocytes. Hum Mutat. 2009 Sep;30(9):1355–64.

15. Howarth R, Yearwood C, Harvey JF. Application of dHPLC for mutation detection of the fibrillin-1 gene for the diagnosis of Marfan syndrome in a National Health Service Laboratory. Genet Test. 2007;11(2):146–52.

16. Loeys B, Nuytinck L, Delvaux I, De Bie S, De Paepe A. Genotype and phenotype analysis of 171 patients referred for molecular study of the fibrillin-1 gene FBN1 because of suspected Marfan syndrome. Arch Intern Med. 2001 Nov 12;161(20):2447–54.

17. Tjeldhorn L, Rand-Hendriksen S, Gervin K, Brandal K, Inderhaug E, Geiran O, et al. Rapid and efficient FBN1 mutation detection using automated sample preparation and direct sequencing as the primary strategy. Genet Test. 2006;10(4):258–64.

18. Hayward C, Porteous ME, Brock DJ. Mutation screening of all 65 exons of the fibrillin-1 gene in 60 patients with Marfan syndrome: report of 12 novel mutations. Hum Mutat. 1997;10(4):280–9.

19. Robinson PN, Booms P, Katzke S, Ladewig M, Neumann L, Palz M, et al. Mutations of FBN1 and genotype-phenotype correlations in Marfan syndrome and related fibrillinopathies. Hum Mutat. 2002 Sep;20(3):153–61.

20. Ståhl-Hallengren C, Ukkonen T, Kainulainen K, Kristofersson U, Saxne T, Tornqvist K, et al. An extra cysteine in one of the non-calcium-binding epidermal growth factor-like motifs of the FBN1 polypeptide is connected to a novel variant of Marfan syndrome. J Clin Invest. 1994 Aug;94(2):709–13.

21. Arbustini E, Grasso M, Ansaldi S, Malattia C, Pilotto A, Porcu E, et al. Identification of sixty-two novel and twelve known FBN1 mutations in eighty-one unrelated probands with Marfan syndrome and other fibrillinopathies. Hum Mutat. 2005 Nov;26(5):494.

22. Zadeh N, Bernstein JA, Niemi AK, Dugan S, Kwan A, Liang D, et al. Ectopia lentis as the presenting and primary feature in Marfan syndrome. Am J Med Genet A. 2011 Nov;155A(11):2661–8.

23. Comeglio P, Johnson P, Arno G, Brice G, Evans A, Aragon-Martin J, et al. The importance of mutation detection in Marfan syndrome and Marfan-related disorders: report of 193 FBN1 mutations. Hum Mutat. 2007 Sep;28(9):928.

24. Haji-Seyed-Javadi R, Jelodari-Mamaghani S, Paylakhi SH, Yazdani S, Nilforushan N, Fan J-B, et al. LTBP2 mutations cause Weill-Marchesani and Weill-Marchesani-like syndrome and affect disruptions in the extracellular matrix. Hum Mutat. 2012 Aug;33(8):1182–7.

25. Nijbroek G, Sood S, McIntosh I, Francomano CA, Bull E, Pereira L, et al. Fifteen novel FBN1 mutations causing Marfan syndrome detected by heteroduplex analysis of genomic amplicons. Am J Hum Genet. 1995 Jul;57(1):8–21.

26. Robinson DO, Lin F, Lyon M, Raponi M, Cross E, White HE, et al. Systematic screening of FBN1 gene unclassified missense variants for splice abnormalities. Clin Genet. 2012 Sep;82(3):223–31.

27. Biggin A, Holman K, Brett M, Bennetts B, Adès L. Detection of thirty novel FBN1 mutations in patients with Marfan syndrome or a related fibrillinopathy. Hum Mutat. 2004 Jan;23(1):99.

28. Rommel K, Karck M, Haverich A, von Kodolitsch Y, Rybczynski M, Müller G, et al. Identification of 29 novel and nine recurrent fibrillin-1 (FBN1) mutations and genotype-phenotype correlations in 76 patients with Marfan syndrome. Hum Mutat. 2005 Dec;26(6):529–39.

29. Comeglio P, Evans AL, Brice G, Cooling RJ, Child AH. Identification of FBN1 gene mutations in patients with ectopia lentis and marfanoid habitus. Br J Ophthalmol. 2002 Dec;86(12):1359–62.

30. Collod-Béroud G, Béroud C, Adès L, Black C, Boxer M, Brock DJ, et al. Marfan Database (second edition): software and database for the analysis of mutations in the human FBN1 gene. Nucleic Acids Res. 1997 Jan 1;25(1):147–50.

31. Milla E, Leszczynska A, Rey A, Navarro M, Larena C. Novel FBN1 mutation causes Marfan syndrome with bilateral ectopia lentis and refractory glaucoma. Eur J Ophthalmol. 2012 Aug;22(4):667–9.

32. Harakalova M, Nijman IJ, Medic J, Mokry M, Renkens I, Blankensteijn JD, et al. Genomic DNA pooling strategy for next-generation sequencing-based rare variant discovery in abdominal aortic aneurysm regions of interest-challenges and limitations. J Cardiovasc Transl Res. 2011 Jun;4(3):271–80.

33. Liu WO, Oefner PJ, Qian C, Odom RS, Francke U. Denaturing HPLC-identified novel FBN1 mutations, polymorphisms, and sequence variants in Marfan syndrome and related connective tissue disorders. Genet Test. 1997 1998;1(4):237–42.

34. Van Den Bossche MJA, Van Wallendael KLP, Strazisar M, Sabbe B, Del-Favero J. Co-occurrence of Marfan syndrome and schizophrenia: what can be learned? Eur J Med Genet. 2012 Apr;55(4):252–5.

35. Uyeda T, Takahashi T, Eto S, Sato T, Xu G, Kanezaki R, et al. Three novel mutations of the fibrillin-1 gene and ten single nucleotide polymorphisms of the fibrillin-3 gene in Marfan syndrome patients. J Hum Genet. 2004;49(8):404–7.

36. Voermans N c, Timmermans J, van Alfen N, Pillen S, op den Akker J, Lammens M, et al. Neuromuscular features in Marfan syndrome. Clin Genet. 2009 Jul;76(1):25–37.

37. Dong J, Bu J, Du W, Li Y, Jia Y, Li J, et al. A new novel mutation in FBN1 causes autosomal dominant Marfan syndrome in a Chinese family. Mol Vis. 2012;18:81–6.

38. Matsukawa R, Iida K, Nakayama M, Mukai T, Okita Y, Ando M, et al. Eight novel mutations of the FBN1 gene found in Japanese patients with Marfan syndrome. Hum Mutat. 2001;17(1):71–2.

39. Karttunen L, Raghunath M, Lönnqvist L, Peltonen L. A compound-heterozygous Marfan patient: two defective fibrillin alleles result in a lethal phenotype. Am J Hum Genet. 1994 Dec;55(6):1083–91.

40. Baumgartner C, Mátyás G, Steinmann B, Baumgartner D. Marfan syndrome--a diagnostic challenge caused by phenotypic and genetic heterogeneity. Methods Inf Med. 2005;44(4):487–97.

41. Collod-Béroud G, Béroud C, Ades L, Black C, Boxer M, Brock DJ, et al. Marfan Database (third edition): new mutations and new routines for the software. Nucleic Acids Res. 1998 Jan 1;26(1):229–223.

42. Van Dijk FS, Hamel BC, Hilhorst-Hofstee Y, Mulder BJM, Timmermans J, Pals G, et al. Compound-heterozygous Marfan syndrome. Eur J Med Genet. 2009 Feb;52(1):1–5.

43. Zhao F, Pan X, Zhao K, Zhao C. Two novel mutations of fibrillin-1 gene correlate with different phenotypes of Marfan syndrome in Chinese families. Mol Vis. 2013;19:751–8.

44. Ogawa N, Imai Y, Takahashi Y, Nawata K, Hara K, Nishimura H, et al. Evaluating Japanese patients with the Marfan syndrome using high-throughput microarray-based mutational analysis of fibrillin-1 gene. Am J Cardiol. 2011 Dec 15;108(12):1801–7.

45. Yoo E-H, Woo H, Ki C-S, Lee HJ, Kim D-K, Kang I-S, et al. Clinical and genetic analysis of Korean patients with Marfan syndrome: possible ethnic differences in clinical manifestation. Clin Genet. 2010 Feb;77(2):177–82.

46. Loeys B, De Backer J, Van Acker P, Wettinck K, Pals G, Nuytinck L, et al. Comprehensive molecular screening of the FBN1 gene favors locus homogeneity of classical Marfan syndrome. Hum Mutat. 2004 Aug;24(2):140–6.

47. Mátyás G, De Paepe A, Halliday D, Boileau C, Pals G, Steinmann B. Evaluation and application of denaturing HPLC for mutation detection in Marfan syndrome: Identification of 20 novel mutations and two novel polymorphisms in the FBN1 gene. Hum Mutat. 2002 Apr;19(4):443–56.

48. Chikumi H, Yamamoto T, Ohta Y, Nanba E, Nagata K, Ninomiya H, et al. Fibrillin gene (FBN1) mutations in Japanese patients with Marfan syndrome. J Hum Genet. 2000;45(2):115–8.

49. Comeglio P, Evans AL, Brice GW, Child AH. Detection of six novel FBN1 mutations in British patients affected by Marfan syndrome. Hum Mutat. 2001 Sep;18(3):251.

50. Piersall LD, Dietz HC, Hall BD, Cadle RG, Pyeritz RE, Francomano CA, et al. Substitution of a cysteine residue in a non-calcium binding, EGF-like domain of fibrillin segregates with the Marfan syndrome in a large kindred. Hum Mol Genet. 1994 Jun;3(6):1013–4.

51. Tang S, Hoshida H, Kamisago M, Yagi H, Momma K, Matsuoka R. Phenotype-genotype correlation in a patient with co-occurrence of Marfan and LEOPARD syndromes. Am J Med Genet A. 2009 Oct;149A(10):2216–9.

52. Hayward C, Brock DJ. Fibrillin-1 mutations in Marfan syndrome and other type-1 fibrillinopathies. Hum Mutat. 1997;10(6):415–23.

53. Schrijver I, Liu W, Brenn T, Furthmayr H, Francke U. Cysteine substitutions in epidermal growth factor-like domains of fibrillin-1: distinct effects on biochemical and clinical phenotypes. Am J Hum Genet. 1999 Oct;65(4):1007–20.

54. Li D, Yu J, Gu F, Pang X, Ma X, Li R, et al. The roles of two novel FBN1 gene mutations in the genotype-phenotype correlations of Marfan syndrome and ectopia lentis patients with marfanoid habitus. Genet Test. 2008 Jun;12(2):325–30.

55. El-Aleem AA, Karck M, Haverich A, Schmidtke J, Arslan-Kirchner M. Identification of 9 novel FBN1 mutations in German patients with Marfan syndrome. Hum Mutat. 1999 Aug 19;14(2):181.

56. Rand-Hendriksen S, Tjeldhorn L, Lundby R, Semb SO, Offstad J, Andersen K, et al. Search for correlations between FBN1 genotype and complete Ghent phenotype in 44 unrelated Norwegian patients with Marfan syndrome. Am J Med Genet A. 2007 Sep 1;143A(17):1968–77.

57. Kilpatrick MW, Lembessis P, Rose E, Tsipouras P. A novel G to A substitution at nucleotide 1734 of the FBN1 gene predicting a C534Y mutation responsible for marfan syndrome. Hum Hered. 1999 Jun;49(3):176–7.

58. Dietz HC, McIntosh I, Sakai LY, Corson GM, Chalberg SC, Pyeritz RE, et al. Four novel FBN1 mutations: significance for mutant transcript level and EGF-like domain calcium binding in the pathogenesis of Marfan syndrome. Genomics. 1993 Aug;17(2):468–75.

59. Khau Van Kien P, Baux D, Pallares-Ruiz N, Baudoin C, Plancke A, Chassaing N, et al. Missense mutations of conserved glycine residues in fibrillin-1 highlight a potential subtype of cb-EGF-like domains. Hum Mutat. 2010 Jan;31(1):E1021–1042.

60. Booms P, Withers AP, Boxer M, Kaufmann UC, Hagemeier C, Vetter U, et al. A novel de novo mutation in exon 14 of the fibrillin-1 gene associated with delayed secretion of fibrillin in a patient with a mild Marfan phenotype. Hum Genet. 1997 Aug;100(2):195–200.

61. Toudjarska I, Kilpatrick MW, Lembessis P, Carra S, Harton GL, Sisson ME, et al. Novel approach to the molecular diagnosis of Marfan syndrome: application to sporadic cases and in prenatal diagnosis. Am J Med Genet. 2001 Apr 1;99(4):294–302.

62. Halliday DJ, Hutchinson S, Lonie L, Hurst JA, Firth H, Handford PA, et al. Twelve novel FBN1 mutations in Marfan syndrome and Marfan related phenotypes test the feasibility of FBN1 mutation testing in clinical practice. J Med Genet. 2002 Aug;39(8):589–93.

63. Lebreiro A, Martins E, Almeida J, Pimenta S, Bernardes JM, Machado JC, et al. Value of molecular diagnosis in a family with Marfan syndrome and an atypical vascular phenotype. Rev Española Cardiol. 2011 Feb;64(2):151–4.

64. Adès LC, Haan EA, Colley AF, Richard RI. Characterisation of four novel fibrillin-1 (FBN1) mutations in Marfan syndrome. J Med Genet. 1996 Aug;33(8):665–71.

65. Hayward C, Rae AL, Porteous ME, Logie LJ, Brock DJ. Two novel mutations and a neutral polymorphism in EGF-like domains of the fibrillin gene (FBN1): SSCP screening of exons 15-21 in Marfan syndrome patients. Hum Mol Genet. 1994 Feb;3(2):373–5.

66. Summers KM, Xu D, West JA, McGill JJ, Galbraith A, Whight CM, et al. An integrated approach to management of Marfan syndrome caused by an FBN1 exon 18 mutation in an Australian Aboriginal family. Clin Genet. 2004 Jan;65(1):66–9.

67. Micheal S, Khan MI, Akhtar F, Weiss MM, Islam F, Ali M, et al. Identification of a novel FBN1 gene mutation in a large Pakistani family with Marfan syndrome. Mol Vis. 2012;18:1918–26.

68. Lledó B, Ten J, Galán FM, Bernabeu R. Preimplantation genetic diagnosis of Marfan syndrome using multiple displacement amplification. Fertil Steril. 2006 Oct;86(4):949–55.

69. Ganesh A, Smith C, Chan W, Unger S, Quercia N, Godfrey M, et al. Immunohistochemical evaluation of conjunctival fibrillin-1 in Marfan syndrome. Arch Ophthalmol. 2006 Feb;124(2):205–9.

70. Tynan K, Comeau K, Pearson M, Wilgenbus P, Levitt D, Gasner C, et al. Mutation screening of complete fibrillin-1 coding sequence: report of five new mutations, including two in 8-cysteine domains. Hum Mol Genet. 1993 Nov;2(11):1813–21.

71. Rommel K, Karck M, Haverich A, Schmidtke J, Arslan-Kirchner M. Mutation screening of the fibrillin-1 (FBN1) gene in 76 unrelated patients with Marfan syndrome or Marfanoid features leads to the identification of 11 novel and three previously reported mutations. Hum Mutat. 2002 Nov;20(5):406–7.

72. Kielty CM, Rantamäki T, Child AH, Shuttleworth CA, Peltonen L. Cysteine-to-arginine point mutation in a “hybrid” eight-cysteine domain of FBN1: consequences for fibrillin aggregation and microfibril assembly. J Cell Sci. 1995 Mar;108 ( Pt 3):1317–23.

73. Judge DP, Biery NJ, Dietz HC. Characterization of microsatellite markers flanking FBN1: utility in the diagnostic evaluation for Marfan syndrome. Am J Med Genet. 2001 Feb 15;99(1):39–47.

74. Hilhorst-Hofstee Y, Rijlaarsdam MEB, Scholte AJHA, Swart-van den Berg M, Versteegh MIM, van der Schoot-van Velzen I, et al. The clinical spectrum of missense mutations of the first aspartic acid of cbEGF-like domains in fibrillin-1 including a recessive family. Hum Mutat. 2010 Dec;31(12):E1915–1927.

75. Collod-Béroud G, Lackmy-Port-Lys M, Jondeau G, Mathieu M, Maingourd Y, Coulon M, et al. Demonstration of the recurrence of Marfan-like skeletal and cardiovascular manifestations due to germline mosaicism for an FBN1 mutation. Am J Hum Genet. 1999 Sep;65(3):917–21.

76. Karttunen L, Ukkonen T, Kainulainen K, Syvänen AC, Peltonen L. Two novel fibrillin-1 mutations resulting in premature termination codons but in different mutant transcript levels and clinical phenotypes. Hum Mutat. 1998;Suppl 1:S34–37.

77. Yuan B, Thomas JP, von Kodolitsch Y, Pyeritz RE. Comparison of heteroduplex analysis, direct sequencing, and enzyme mismatch cleavage for detecting mutations in a large gene, FBN1. Hum Mutat. 1999;14(5):440–6.

78. Qin Y, Yan J, Simpson JL, Gu HF, Wang L-C, Chen Z-J. Novel non-synonymous mutation in the transforming growth factor beta binding protein-like (TB) domain of the fibrillin-1 (FBN1) gene in a Han Chinese family with Marfan syndrome (MFS). Neuro Endocrinol Lett. 2007 Oct;28(5):629–32.

79. Lebreiro A, Martins E, Cruz C, Almeida J, Pimenta S, Bernardes M, et al. [Genotypic characterization of a Portuguese population of Marfan syndrome patients]. Rev Port Cardiol Orgão Of Soc Port Cardiol Port J Cardiol Off J Port Soc Cardiol. 2011 Jul;30(7-8):649–54.

80. Kainulainen K, Karttunen L, Puhakka L, Sakai L, Peltonen L. Mutations in the fibrillin gene responsible for dominant ectopia lentis and neonatal Marfan syndrome. Nat Genet. 1994 Jan;6(1):64–9.

81. Evangelisti L, Lucarini L, Attanasio M, Lapini I, Giusti B, Porciani C, et al. A single heterozygous nucleotide substitution displays two different altered mechanisms in the FBN1 gene of five Italian Marfan patients. Eur J Med Genet. 2010 Oct;53(5):299–302.

82. Lo IF, Wong RM, Lam FW, Tong TM, Lam ST. Missense mutations of the fibrillin-1 gene in two Chinese patients with severe Marfan syndrome. Chin Med J (Engl). 2001 May;114(5):473–6.

83. Ng DK, Chau KW, Black C, Thomas TM, Mak KL, Boxer M. Neonatal Marfan syndrome: a case report. J Paediatr Child Health. 1999 Jun;35(3):321–3.

84. Wang M, Wang JY, Cisler J, Imaizumi K, Burton BK, Jones MC, et al. Three novel fibrillin mutations in exons 25 and 27: classic versus neonatal Marfan syndrome. Hum Mutat. 1997;9(4):359–62.

85. Stheneur C, Faivre L, Collod-Béroud G, Gautier E, Binquet C, Bonithon-Kopp C, et al. Prognosis factors in probands with an FBN1 mutation diagnosed before the age of 1 year. Pediatr Res. 2011 Mar;69(3):265–70.

86. Lönnqvist L, Karttunen L, Rantamäki T, Kielty C, Raghunath M, Peltonen L. A point mutation creating an extra N-glycosylation site in fibrillin-1 results in neonatal Marfan syndrome. Genomics. 1996 Sep 15;36(3):468–75.

87. Putnam EA, Cho M, Zinn AB, Towbin JA, Byers PH, Milewicz DM. Delineation of the Marfan phenotype associated with mutations in exons 23-32 of the FBN1 gene. Am J Med Genet. 1996 Mar 29;62(3):233–42.

88. Wang M, Kishnani P, Decker-Phillips M, Kahler SG, Chen YT, Godfrey M. Double mutant fibrillin-1 (FBN1) allele in a patient with neonatal Marfan syndrome. J Med Genet. 1996 Sep;33(9):760–3.

89. Sutherell J, Zarate Y, Tinkle BT, Markham LW, Cripe LH, Hyland JC, et al. Novel fibrillin 1 mutation in a case of neonatal Marfan syndrome: the increasing importance of early recognition. Congenit Heart Dis. 2007 Oct;2(5):342–6.

90. Cui Y, Zhao H, Liu Z, Liu C, Luan J, Zhou X, et al. A systematic review of genetic skeletal disorders reported in Chinese biomedical journals between 1978 and 2012. Orphanet J Rare Dis. 2012;7:55.

91. Tiecke F, Katzke S, Booms P, Robinson PN, Neumann L, Godfrey M, et al. Classic, atypically severe and neonatal Marfan syndrome: twelve mutations and genotype-phenotype correlations in FBN1 exons 24-40. Eur J Hum Genet EJHG. 2001 Jan;9(1):13–21.

92. Dietz HC, Cutting GR, Pyeritz RE, Maslen CL, Sakai LY, Corson GM, et al. Marfan syndrome caused by a recurrent de novo missense mutation in the fibrillin gene. Nature. 1991 Jul 25;352(6333):337–9.

93. Milewicz DM, Michael K, Fisher N, Coselli JS, Markello T, Biddinger A. Fibrillin-1 (FBN1) mutations in patients with thoracic aortic aneurysms. Circulation. 1996 Dec 1;94(11):2708–11.

94. Perez AB, Pereira LV, Brunoni D, Zatz M, Passos-Bueno MR. Identification of 8 new mutations in Brazilian families with Marfan syndrome. Mutations in brief no. 211. Online. Hum Mutat. 1999;13(1):84.

95. Valiev RR, Khusainova RI, Kutuev IA, Khusnutdinova EK. [AFBN1 gene in patients with Marfan syndrome]. Mol Biol (Mosk). 2006 Dec;40(6):1021–30.

96. Pepe G, Giusti B, Attanasio M, Comeglio P, Porciani MC, Giurlani L, et al. A major involvement of the cardiovascular system in patients affected by Marfan syndrome: novel mutations in fibrillin 1 gene. J Mol Cell Cardiol. 1997 Jul;29(7):1877–84.

97. Rantamäki T, Kaitila I, Syvänen AC, Lukka M, Peltonen L. Recurrence of Marfan syndrome as a result of parental germ-line mosaicism for an FBN1 mutation. Am J Hum Genet. 1999 Apr;64(4):993–1001.

98. Derbent M, Anuk D, Tarcan A, Varan B, Gurakan B, Tokel K. Functional pulmonary atresia in a patient with neonatal Marfan syndrome caused by a c.3602G>A mutation in exon 29 of the FBN1 gene. Clin Dysmorphol. 2008 Apr;17(2):127–8.

99. Hewett DR, Lynch JR, Child A, Sykes BC. A new missense mutation of fibrillin in a patient with Marfan syndrome. J Med Genet. 1994 Apr;31(4):338–9.

100. Meng B, Li H, Yang T, Huang S, Sun X, Yuan H. Identification of a novel FBN1 gene mutation in a Chinese family with Marfan syndrome. Mol Vis. 2011;17:2421–7.

101. Ter Heide H, Schrander-Stumpel CTRM, Pals G, Delhaas T. Neonatal Marfan syndrome: clinical report and review of the literature. Clin Dysmorphol. 2005 Apr;14(2):81–4.

102. Dietz HC, Pyeritz RE. Mutations in the human gene for fibrillin-1 (FBN1) in the Marfan syndrome and related disorders. Hum Mol Genet. 1995;4 Spec No:1799–809.

103. Oh MR, Kim JS, Beck NS, Yoo HW, Lee HJ, Kohsaka T, et al. Six novel mutations of the fibrillin-1 gene in Korean patients with Marfan syndrome. Pediatr Int Off J Jpn Pediatr Soc. 2000 Oct;42(5):488–91.

104. Montgomery RA, Geraghty MT, Bull E, Gelb BD, Johnson M, McIntosh I, et al. Multiple molecular mechanisms underlying subdiagnostic variants of Marfan syndrome. Am J Hum Genet. 1998 Dec;63(6):1703–11.

105. Youil R, Toner TJ, Bull E, Bailey AL, Earl CD, Dietz HC, et al. Enzymatic mutation detection (EMD) of novel mutations (R565X and R1523X) in the FBN1 gene of patients with Marfan syndrome using T4 endonuclease VII. Hum Mutat. 2000 Jul;16(1):92–3.

106. Halliday D, Hutchinson S, Kettle S, Firth H, Wordsworth P, Handford PA. Molecular analysis of eight mutations in FBN1. Hum Genet. 1999 Dec;105(6):587–97.

107. Sheikhzadeh S, Kade C, Keyser B, Stuhrmann M, Arslan-Kirchner M, Rybczynski M, et al. Analysis of phenotype and genotype information for the diagnosis of Marfan syndrome. Clin Genet. 2012 Sep;82(3):240–7.

108. Dietz HC, Saraiva JM, Pyeritz RE, Cutting GR, Francomano CA. Clustering of fibrillin (FBN1) missense mutations in Marfan syndrome patients at cysteine residues in EGF-like domains. Hum Mutat. 1992;1(5):366–74.

109. Huang X, Wu Y, Chen F, Huang Y, Ma X, Chen T. [Two novel mutations in fibrillin-1 gene of Marfan syndrome]. Zhonghua Yi Xue Yi Chuan Xue Za Zhi Zhonghua Yixue Yichuanxue Zazhi Chin J Med Genet. 2004 Dec;21(6):562–5.

110. Zhao L, Liang T, Xu J, Lin H, Li D, Qi Y. Two novel FBN1 mutations associated with ectopia lentis and marfanoid habitus in two Chinese families. Mol Vis. 2009;15:826–32.

111. Hewett DR, Lynch JR, Smith R, Sykes BC. A novel fibrillin mutation in the Marfan syndrome which could disrupt calcium binding of the epidermal growth factor-like module. Hum Mol Genet. 1993 Apr;2(4):475–7.

112. Ng PC, Henikoff S. Accounting for human polymorphisms predicted to affect protein function. Genome Res. 2002 Mar;12(3):436–46.

113. Díaz de Bustamante A, Ruiz-Casares E, Darnaude MT, Perucho T, Martínez-Quesada G. Phenotypic variability in Marfan syndrome in a family with a novel nonsense FBN1 gene mutation. Rev Española Cardiol Engl Ed. 2012 Apr;65(4):380–1.

114. Katzke S, Booms P, Tiecke F, Palz M, Pletschacher A, Türkmen S, et al. TGGE screening of the entire FBN1 coding sequence in 126 individuals with marfan syndrome and related fibrillinopathies. Hum Mutat. 2002 Sep;20(3):197–208.

115. Hilhorst-Hofstee Y, Kroft LJM, Pals G, van Vugt JPP, Overweg-Plandsoen WCG. Intracranial hypertension in 2 children with marfan syndrome. J Child Neurol. 2008 Aug;23(8):954–5.

116. Loeys B, Nuytinck L, Van Acker P, Walraedt S, Bonduelle M, Sermon K, et al. Strategies for prenatal and preimplantation genetic diagnosis in Marfan syndrome (MFS). Prenat Diagn. 2002 Jan;22(1):22–8.

117. Palz M, Tiecke F, Booms P, Göldner B, Rosenberg T, Fuchs J, et al. Clustering of mutations associated with mild Marfan-like phenotypes in the 3’ region of FBN1 suggests a potential genotype-phenotype correlation. Am J Med Genet. 2000 Mar 20;91(3):212–21.

118. Song Y-H, Kim G-H, Yoo H-W, Kim J-B. Novel de novo nonsense mutation of FBN1 gene in a patient with Marfan syndrome. J Genet. 2012 Aug;91(2):233–5.

119. Hogue J, Lee C, Jelin A, Strecker M, Cox V, Slavotinek A. Homozygosity for a FBN1 missense mutation causes a severe Marfan syndrome phenotype. Clin Genet. 2013 Oct 28;84(4):392–3.

120. Kainulainen K, Sakai LY, Child A, Pope FM, Puhakka L, Ryhänen L, et al. Two mutations in Marfan syndrome resulting in truncated fibrillin polypeptides. Proc Natl Acad Sci U S A. 1992 Jul 1;89(13):5917–21.

121. Hayward C, Porteous ME, Brock DJ. Identification of a novel nonsense mutation in the fibrillin gene (FBN1) using nonisotopic techniques. Hum Mutat. 1994;3(2):159–62.

122. Gao L, Zhang L, Song L, Wang H, Chang Q, Wu Y, et al. Identification of a novel lethal fibrillin-1 gene mutation in a Chinese Marfan family and correlation of 3’ fibrillin-1 gene mutations with phenotype. Chin Med J (Engl). 2010 Oct;123(20):2874–8.

123. Stheneur C, Collod-Béroud G, Faivre L, Gouya L, Sultan G, Le Parc J-M, et al. Identification of 23 TGFBR2 and 6 TGFBR1 gene mutations and genotype-phenotype investigations in 457 patients with Marfan syndrome type I and II, Loeys-Dietz syndrome and related disorders. Hum Mutat. 2008 Nov;29(11):E284–295.

124. Chung BH-Y, Lam ST-S, Tong TM-F, Li SY-H, Lun K-S, Chan DH-C, et al. Identification of novel FBN1 and TGFBR2 mutations in 65 probands with Marfan syndrome or Marfan-like phenotypes. Am J Med Genet A. 2009 Jul;149A(7):1452–9.

125. Mizuguchi T, Collod-Beroud G, Akiyama T, Abifadel M, Harada N, Morisaki T, et al. Heterozygous TGFBR2 mutations in Marfan syndrome. Nat Genet. 2004 Aug;36(8):855–60.

126. Singh KK, Rommel K, Mishra A, Karck M, Haverich A, Schmidtke J, et al. TGFBR1 and TGFBR2 mutations in patients with features of Marfan syndrome and Loeys-Dietz syndrome. Hum Mutat. 2006 Aug;27(8):770–7.

127. Disabella E, Grasso M, Marziliano N, Ansaldi S, Lucchelli C, Porcu E, et al. Two novel and one known mutation of the TGFBR2 gene in Marfan syndrome not associated with FBN1 gene defects. Eur J Hum Genet EJHG. 2006 Jan;14(1):34–8.

128. Zhang L, Gao L-G, Zhang M, Zhou X-L. Genotype-phenotype analysis of F-helix mutations at the kinase domain of TGFBR2, including a type 2 Marfan syndrome familial study. Mol Vis. 2012;18:55–63.

129. Attias D, Stheneur C, Roy C, Collod-Béroud G, Detaint D, Faivre L, et al. Comparison of clinical presentations and outcomes between patients with TGFBR2 and FBN1 mutations in Marfan syndrome and related disorders. Circulation. 2009 Dec 22;120(25):2541–9.

130. Mátyás G, Arnold E, Carrel T, Baumgartner D, Boileau C, Berger W, et al. Identification and in silico analyses of novel TGFBR1 and TGFBR2 mutations in Marfan syndrome-related disorders. Hum Mutat. 2006 Aug;27(8):760–9.

131. Singh KK, Rommel K, Mishra A, Karck M, Haverich A, Schmidtke J, et al. TGFBR1 and TGFBR2 mutations in patients with features of Marfan syndrome and Loeys-Dietz syndrome. Hum Mutat. 2006 Aug;27(8):770–7.

132. Mátyás G, Arnold E, Carrel T, Baumgartner D, Boileau C, Berger W, et al. Identification and in silico analyses of novel TGFBR1 and TGFBR2 mutations in Marfan syndrome-related disorders. Hum Mutat. 2006 Aug;27(8):760–9.

133. Stheneur C, Collod-Béroud G, Faivre L, Gouya L, Sultan G, Le Parc J-M, et al. Identification of 23 TGFBR2 and 6 TGFBR1 gene mutations and genotype-phenotype investigations in 457 patients with Marfan syndrome type I and II, Loeys-Dietz syndrome and related disorders. Hum Mutat. 2008 Nov;29(11):E284–295.
