# Supplementary material for: New population-based exome data question the pathogenicity of some genetic variants previously associated with Marfan syndrome
Source: BMC Genet. 2014 Jun 18;15:74. doi: 10.1186/1471-2156-15-74 (PMC4070351; doi:10.1186/1471-2156-15-74)
Supplement: Additional file 3: Table S2 — Clinical data of FBN-1 ESP-positive variants. [file 1471-2156-15-74-S3.docx]

**Additional file 3 clinical data of FBN-1 ESP-positive variants**

| **Nucleotide Change**  **(IVS #)** | **Protein change** | **Sex** | **age** | **Familial (+)**  **Non proven(np)**  ***De novo*** | **Cardiovascular system** | **Ocular System** | **Skeletal System** | **Nervous** | **Skin** | **Lung** | Diagnostic MFS |
| --- | --- | --- | --- | --- | --- | --- | --- | --- | --- | --- | --- |
| c.59A>G | p.Y20C | M | 14 | + | - | - | Involv | / | - | pnx | MFS |
| c.1027G>A | p.G343R | NA | NA | NA | NA | NA | NA | NA | NA | NA | MFS |
| c.1345G>A | p.V449I | M | 15 | Not proven | + | - | - | / | / | / | MFS |
| c.2056G>A | p.A686T | NA | NA | NA | - | - | - | NA | NA | NA | IM |
| c.2927G>A | p.R976H | NA | 22 | NA | NA | NA | NA | NA | NA | NA | MFS |
| c.3058A>G | p.T1020A | F | 3 | + | - | NA | + | NA | NA | NA | MFS |
| c.3422C>T | p.P1141L | NA | NA | NA | NA | NA | NA | NA | NA | NA | MFS |
| c.3509G>A | p.R1170H | NA | NA | NA | NA | NA | NA | NA | NA | NA | MFS |
| c.3797A>T | p.Y1266F | NA | NA | NA | NA | NA | NA | NA | NA | NA | MFS |
| c.3845A>G | p.N1282S | NA | NA | NA | NA | NA | NA | NA | NA | NA | MFS |
| c.4270C>G | p.P1424A | NA | NA | NA | - | - | + | - | - | - | MFS |
| c.6055G>A | p.E2019K | NA | NA | + | NA | NA | NA | NA | NA | NA | MFS |
| c.6700G>A | p.V2234M | NA | NA | NA | NA | NA | NA | NA | NA | NA | MFS |
| c.7241G>A | p.R2414Q | 6 | F | + | + | - | - | - | - | - | MFS |
| c.7379A>G | p.K2460R | NA | NA | NA | NA | NA | NA | NA | NA | NA | MFS |
| c.7660C>T | p.R2554W | NA | 70 | + | + | - | - | - | - | - | IM |
| c.7661G>A | p.R2554Q | NA | NA | - | NA | NA | NA | NA | NA | NA | MFS |
| c.7702G>A | p.V2568M | NA | NA | NA | NA | NA | NA | NA | NA | NA | MFS |
| c.7846A>G | p.I2616V | NA | NA | NA | NA | NA | NA | NA | NA | NA | MFS |
| c.7852G>A | p.G2618R | NA | NA | NA | NA | NA | NA | NA | NA | NA | MFS |
| c.8081G>A | p.R2694Q | NA | NA | NA | NA | NA | NA | NA | NA | NA | MFS |
| c.8176C>T | p.R2726W | M | 13 | + | - | NA | + | NA | NA | NA | MFS |
| c.8494A>G | p.S2832G | NA | NA | NA | NA | NA | NA | NA | NA | NA | MFS |

M=male; F=Female; np = not proven; Involv = involvement; + skin = minor criteria; pnx = spontaneous pneumo-thorax; nd = not done; **/** = not done and not planned in our；NA=Not Available; MFS=Marfan syndrome; IM=incomplete MFS
