# Supplementary material for: New population-based exome data question the pathogenicity of some genetic variants previously associated with Marfan syndrome
Source: BMC Genet. 2014 Jun 18;15:74. doi: 10.1186/1471-2156-15-74 (PMC4070351; doi:10.1186/1471-2156-15-74)

Additional file 4: Figure S1: Percentage of variants predicted to be pathogenic with four In silico tools prediction on variants present and not present in ESP database. Differences in proportions of variants predicted to be damaging for those variants present in ESP versus variants not present in ESP. Furthermore, variants with low frequency (rare non-synonymous variants (<=2)) in the ESP is also shown.


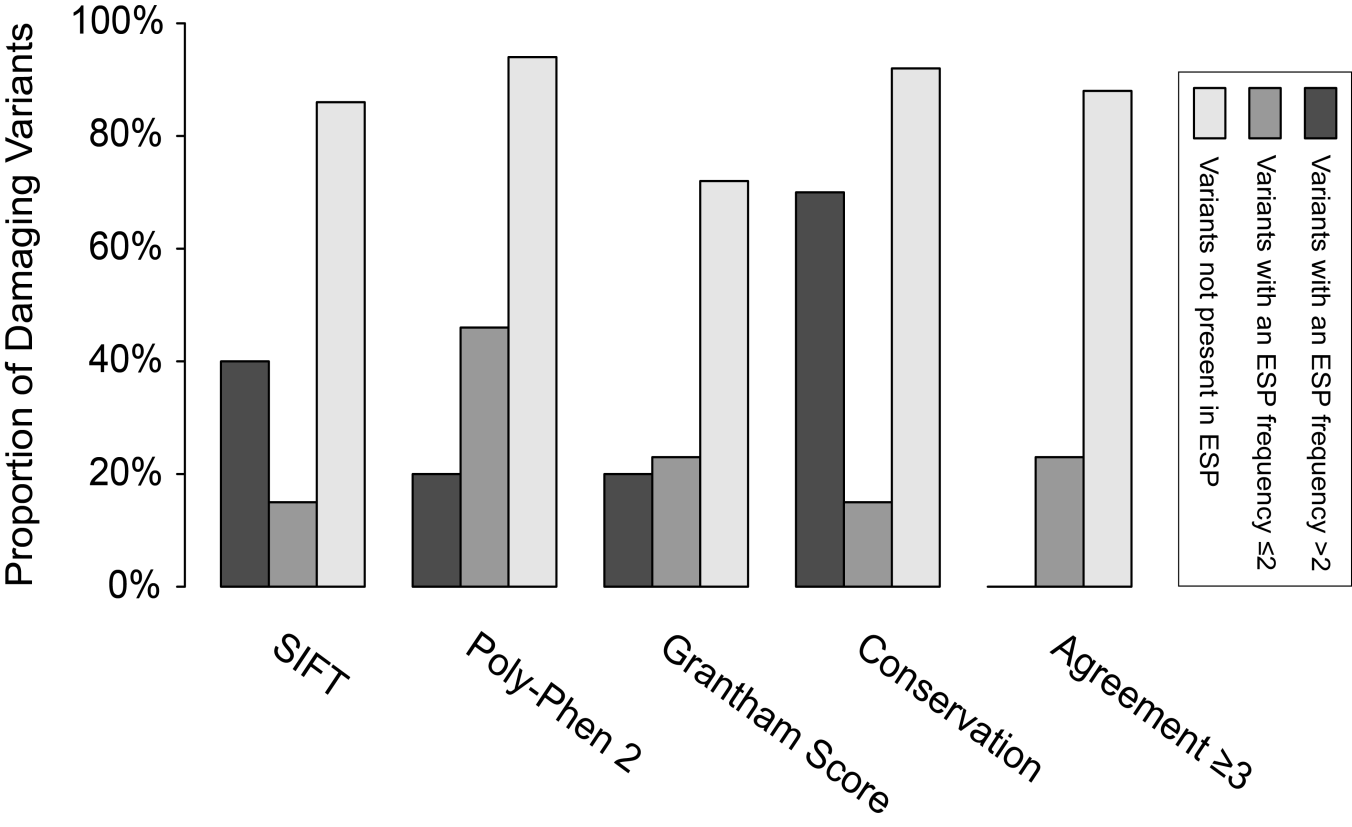

Supplement: Additional file 4: Figure S1 — Percentage of variants predicted to be pathogenic with four In silico tools prediction on variants present and not present in ESP database. Differences in proportions of variants predicted to be damaging for those variants present in ESP versus variants not present in ESP. Furthermore, variants with low frequency (rare non-synonymous variants (<=2)) in the ESP is also shown. [file 1471-2156-15-74-S4.docx]
